# Beliefs and behaviors associated with the first named heat wave in Seville Spain 2022

**DOI:** 10.1038/s41598-024-59430-8

**Published:** 2024-04-20

**Authors:** Aaron Metzger, Yuval Baharav, Lilly Nichols, Megan Finke, Breahnna Saunders, Peter Mitchell, Gregory A. Wellenius, Kathy Baughman McLeod, Kurt Shickman

**Affiliations:** 1 Marketing for Change, Alexandria, VA USA; 2 Adrienne Arsht-Rockefeller Foundation Resilience Center at the Atlantic Council, Washington, DC USA; 3 Center for Climate and Health, Boston University School of Public Health, Boston, MA USA

**Keywords:** Climate-change impacts, Climate-change adaptation, Psychology and behaviour

## Abstract

Heat waves pose a substantial and increasing risk to public health. Heat health early warning systems (HHEWSs) and response plans are increasingly being adopted to alert people to the health risks posed by days of extreme heat and recommend protective behaviors. However, evidence regarding the effectiveness of HHEWSs remains limited. We examined the impact of heat wave naming on heat-related beliefs and behaviors to ascertain the potential effectiveness of heat wave naming as a heat health risk communication and management tool. Specifically, we surveyed members of the public exposed to the proMETEO Sevilla HHEWS messaging campaign which in the summer of 2022 applied a name to heat waves considered to pose the greatest risk to public health. During the heat season we evaluated, the proMETEO Sevilla HHEWS campaign applied a name to one heat wave, heat wave “*Zoe*”. Our analysis of the post-survey of 2022 adults indicated that the 6% of participants who recalled the name *Zoe* unaided reported greater engagement in heat wave safety behaviors and more positive beliefs about naming heat waves and their local governments’ heat wave response. These results provide initial evidence for potential utility in naming heat waves as part of HHEWSs and HAPs.

## Introduction

Extreme heat events, or heat waves, represent a major public health risk, thought to be responsible for nearly half a million deaths annually worldwide^1^. In an average year, more deaths are associated with heat waves than with any other meteorological event including hurricanes, tornadoes, and snowstorms^2,3^. After the devastating 2003 heat wave in Europe, the WHO (World Health Organization) and WMO (World Meteorological Organization) urged countries to develop heat early warning systems and heat health adaptation plans^4^. In response to this recognized threat, communities around the globe are implementing and/or refining heat early warning systems and response plans, often referred to as heat action plans (HAPs)^5^. HAPs often feature messaging about the potential adverse health impacts of extreme heat events, who might be at particularly high risk, and actions individuals can take to better protect themselves^5–7^. Despite substantial investments in optimizing HAPs, extreme heat events remain a significant threat to public health. For example, an estimated 60,000 excess deaths were attributable to heat in Europe during summer 2022^8^, despite significant investments and improvements in the population heat response following deadly heat waves in 2003 and more recently^9,10^.

The research presented here aligns with global efforts to improve and broaden the use of early warning systems for weather disaster risks. United Nations Secretary General António Gutteres recognized the global need to improve access to weather-related warnings in a 2022 statement announcing the formation of an Early Warnings for All initiative (EW4All) with leadership from the World Meteorological Organization, United Nations Development Program, International Federation of the Red Cross/Red Crescent and others. The initiative’s 2023–2027 Action Plan calls for investments of $3.1 billion to strengthen disaster risk data management, forecasting, and public engagement. The EW4All Action Plan is built around 4 pillars to increase access, improve technical capacity for forecasting, alignment with global financing and funding priorities, and a people-centered approach to designing and delivering warnings.

Additionally, with support from the Adrienne Arsht-Rockefeller Foundation Resilience Center (Arsht-Rock), HHEWS (Heat Health Early Warning Systems) are being piloted throughout Greece with the National Observatory of Athens, and in Seville, Spain^11,12^. The Seville pilot was called ProMETEO Sevilla, and was implemented in partnership with the University of Seville and the Seville City Council.

**Figure 1:**
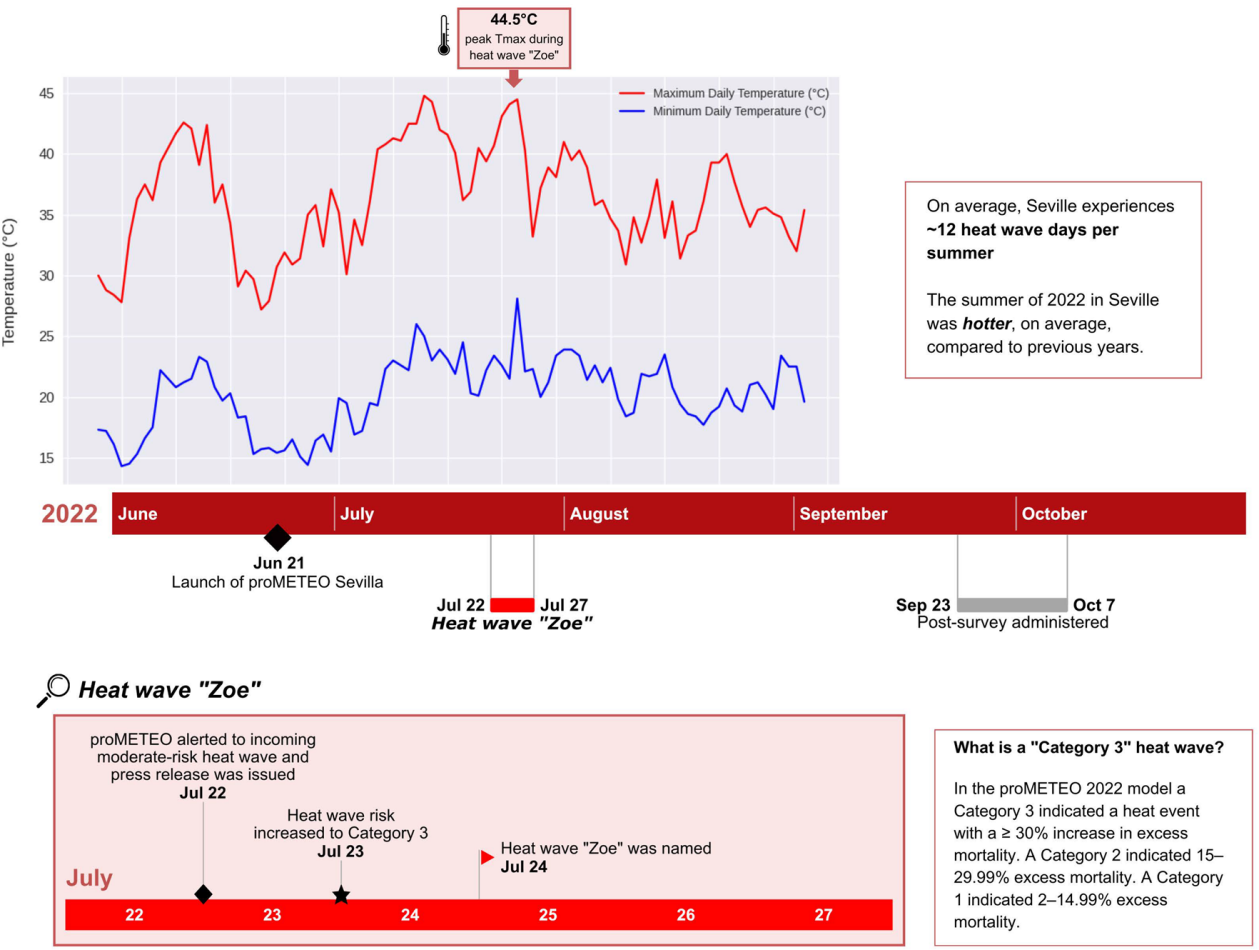
Project timeline during summer 2022 in Southern Spain.

In the proMETEO Sevilla HHEWS, not only are extreme heat events categorized based on their expected health impacts, but names are also attached to those heat events forecast to have the most extreme health impacts. One particularly severe heat wave in Seville in the summer of 2022 triggered the proMETEO HHEWS naming threshold and became the world’s first named heat wave after being given the name *Zoe* (please see Fig. 1 for a timeline of the Seville heat season and proMETEO pilot activities)^13^. News about the naming of heat wave *Zoe* spread across traditional and social media outlets.

The goal of heat wave naming was to increase public awareness and attention to the most deadly heat waves and encourage more individuals to engage in appropriate safety behaviors. However, whether naming heat waves is an effective way to increase awareness of the health risks posed by extreme heat events or alter individual’s behaviors is unknown. Naming weather events is a commonly used method to communicate a weather system’s severity to the public, however the evidence for whether naming effectively conveys the severity of a weather event, influences preparation, or encourages behavior uptake to reduce health risks associated with the event, is mixed. Although meteorological agencies typically name severe tropical cyclones (e.g., hurricanes or typhoons), naming of other extreme weather events, including heat waves, is a novel idea that has raised some concerns^14^. For instance, in September 2022, the WMO published a report raising important concerns about heat wave naming, including highlighting the lack of real-world evidence about the effectiveness or risks associated with this approach^15^. Accordingly, this study seeks to examine if naming a heat wave had a measurable impact on the Seville public’s recall, awareness, beliefs, and behaviors in summer 2022. We hope that this study can be a starting point for a more developed base of literature assessing the validity of heat wave naming as a protective public health intervention. Specifically, we conducted a survey to assess public awareness of the heat wave’s name (recall and awareness), the prevalence of heat health beliefs, and engagement in heat safety behaviors. We subsequently evaluated the associations between awareness and both beliefs and behaviors.

## Methods

### The proMETEO heat wave categorization and naming intervention

Seville is the hottest major metropolitan area in Europe^16^. The elected city government in 2022 supported testing a novel approach to reduce the adverse health impacts of extreme heat events. A partnership between the City of Seville, Arsht-Rock, the University of Seville, and other local partners lead to the development and implementation of a HHEWS, including an effort to publicly name the most dangerous heat waves.The proMETEO Sevilla HHEWS methodology was developed building on the methodology used in the Philadelphia Hot Weather-Health Watch/Warning System initiated in 1995^17^. The proMETEO Sevilla HHEWS leveraged decades of local meteorological and mortality data to build a model to prospectively identify and categorize the impact of extreme heat to human health, resulting in a localized, tiered heatwave categorization model that predicts the risk of excess mortality in upcoming heat days. Heat waves predicted to have the greatest excess mortality were eligible for naming. In July of 2022, a 5-day event in Seville was identified as a category 3 heat wave, the highest category of the proMETEO Sevilla HHEWS, associated with a 30% or greater excess mortality in Seville (please see Fig. 1 for a timeline of the Seville heat season and proMETEO pilot activities)^12^.

The pilot project included Descubre, a Seville-based communications agency selected by the Mayor’s office that developed a digital campaign and a modest media strategy. The proMETEO Sevilla campaign included social media notices and promotion of local and regional news coverage through local partners' social media channels. Messages distributed in advance of and during extreme weather events featured thermometer graphics illustrating the category of heat wave, messages encouraging heat safety behaviors, and additional information about the health effects of climate change (see supplemental materials for examples).

The name *Zoe* was announced on July 24, 2022 and corresponded to the extreme heat event that occurred between July 22 and July 27, 2022. The decision of when to attach a name to a heat wave was jointly made by the system implementation partners—the City Council of Seville, the University of Seville, and the Adrienne Arsht-Rockefeller Foundation Resilience Center. During this event, daytime temperatures exceeded 43 °C (109°F) and were accompanied by high nighttime temperatures^13^. News about the naming of heat wave *Zoe* spread across traditional and social media outlets (see supplemental materials). A full description of the proMETEO messaging campaign is described elsewhere (https://prometeosevilla.com)^18^.

### Study design and procedures

Between September 23 and October 7, 2022, we distributed a survey via a professional recruiter to an online panel of adults aged 25 and older living in the following regions of southern Spain: Badajoz, Caceras, Cordoba, Madrid, Malaga, and Salamanca, and Seville. Cordoba and Seville were the intervention regions where the ProMETEO Sevilla categorization and naming pilot was primarily targeted. The other regions were chosen because they share similar meteorological conditions to Seville and Cordoba during the summer months, but were not specifically targeted by proMETEO Sevilla with messaging about the health hazards of extreme heat or *Zoe*. However, analysis of messaging indicated that unaided recall of the heat wave name was high in the control regions and only slightly lower than the recall in the targeted regions of Seville and Cordoba. This indicates that individuals in the control regions were also exposed to the naming dimension of this campaign at levels that were nearly identical to the targeted region. There are multiple potential reasons for this. Social media posts containing the naming/ranking campaign could have been shared with individuals across the targeted and control regions. However, a more likely explanation is the news coverage of the heat wave naming, which was included across multiple media sources at the regional and even national level. Regardless, because of the high levels of messaging exposure within the control group, a single analytic sample that included both targeted and control regions was utilized for subsequent analysis.

The survey assessed participants’ engagement in heat wave safety behaviors, as well as their beliefs about heat waves. The survey also included measures designed to assess participants’ exposure to and awareness of the proMETEO heat wave categorizing and naming pilot (i.e., whether participants recalled the heat wave name and categorization system).

### Measures

#### Heat wave safety behaviors

Based on previous research^19^, participants were asked, “*What did you do during the last heat wave you remember?”* and were presented with a checklist of eleven behaviors. The measure captures a range of behaviors including *risk avoidance*, *risk reduction,* and *prosocial* behaviors. *Avoidance* heat wave behaviors (5 items) aim to evade extreme heat altogether, such as by spending more time indoors or adjusting work or leisure activities. In contrast, *reduction* heat wave behaviors (3 items) aim to lessen or mitigate the effects of extreme heat, such as by drinking more water or wearing different clothes. *Prosocial* heat wave behaviors (3 items) include helping others to avoid the risks of extreme heat or communicating with others about a heat wave.

#### Heat wave beliefs

The survey also included measures of participants’ beliefs about heat waves. Beliefs in this study are defined by degree agreement with a given condition. For each item, participants indicated their level of agreement on a four-point scale (*strongly disagree, disagree, agree, strongly agree)*. Four heat wave *belief* items assessed beliefs on the potential dangers of extreme heat both for the individual and in general (see supplemental materials for survey instrument), two items measured individual’s self-efficacy to engage in safe heat wave behaviors, and a single item assessed participants’ views of their local government’s efforts to protect citizens during heat waves.

#### ProMETEO Sevilla categorizing and naming pilot and messaging campaign exposure and recall

A series of questions were asked to gauge participants’ awareness of the proMETEO Sevilla heat wave categorization and naming pilot. When asked to recall specifics about the categorization component of the proMETEO campaign, recall was low (15% picked correct categorization system from a list of 6 options, slightly lower than random chance) and was not statistically significantly associated with heat wave safety behavior or attitudes. As a result we focused our analysis on metrics related to the heat wave naming component of the pilot which demonstrated stronger recall.

**Figure 2:**
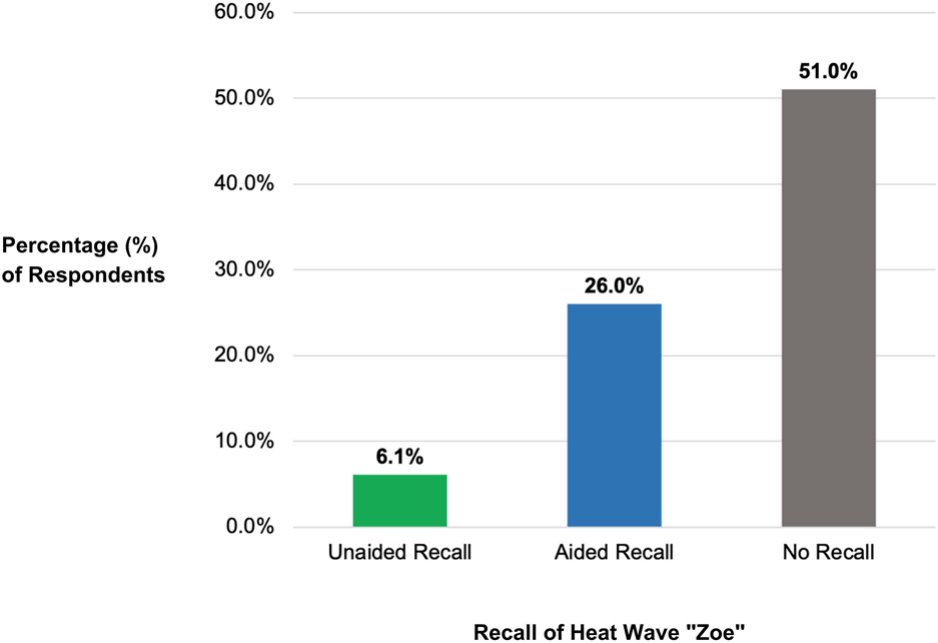
Bar chart depicting percent recall of heat wave “Zoe” among study participants in Southern Spain.

Two categories of awareness of the heat wave name were measured: *unaided* recall and *aided* recall. Participants with *unaided* recall are those who can spontaneously reference specific aspects of the messaging campaign without prompting or help. First, participants were asked how many heat waves they remembered that received a name during the previous summer *(“How many, if any heat waves this summer were named?”* 0, 1, 2, 3 or more*).* Those that recalled that at least one heat wave had been named were given an open-ended response question in which they were asked to write in the name of the heat wave *(“What was/were the name(s) of the heatwave(s)?”)*. Responses were coded for their inclusion of the heat wave name *Zoe*, including alternate spellings. Participants who wrote in *Zoe* were included in the unaided recall category (n = 123, 6.1%)(Fig. 2). As a follow-up, participants were given an aided recall question (*Do you recall a heat wave named “Zoe” this summer? yes/no).* Participants who responded with a “yes” to this question and who had not correctly identified *Zoe* in the unaided recall question were grouped into the *aided* recall group (n = 526, 26%) (Fig. 2). Within marketing research, unaided recall is considered a robust measure of messaging exposure, but it is also a more conservative estimate of recall given the cognitive demands. In contrast, aided recall is less cognitively taxing but it is more prone to response errors^20^.

#### Demographic control variables

Participants were promoted to report a range of demographic variables including age, gender, health status, and monthly income in Euros.

#### Analysis strategy

ANOVAs (Analysis of Variance) and chi-square analyses were used to test for associations between participants’ demographic characteristics and aided and unaided recall. To assess associations between messaging campaign awareness and both engagement in heat wave safety behavior and beliefs about heat waves, a series of hierarchical linear regression models were tested. Each model included participants’ gender, age, monthly income, and self-reported health status as control variables in step 1. Aided and unaided recall were included in step 2. Separate models were run for each category of heat wave safety behavior category (avoidance, reduction, prosocial), and for each separate perception/attitude. A 2-sided *p*-value < 0.05 was considered statistically significant. Analyses were conducted using SPSS v. 29.

## Results

Among the 2022 participants aged 25 to over 82 that completed the online survey, approximately half (53.8%) identified as female. Compared to the overall Spanish population, younger adults (aged 25–44) were overrepresented in the survey (56.7% survey, 36.2% Spanish population), while older adults (65 and older) were underrepresented (6.1% survey, 23.8% Spanish population) (older adults tend to be underrepresented in online surveys^21^). The majority of respondents (70.8%) reported earning between 800 and 2700 euros per month and that their health status was “good” (57.7%) or “very good” (18.3%).

### Awareness of the messaging campaign

When asked if they remembered how many heat waves from the previous summer had been named, 49% indicated that at least one heat wave had been named the previous summer, but only 6% of respondents correctly recalled the name *Zoe* (or a variation spelling) unaided when asked what the heat wave had been named (Fig. 2). An additional 26% reported remembering that a heat wave had been named *Zoe* for the aided recall question (*Do you recall a heat wave named Zoe?)*. In total, 32% of participants indicated that they remembered the heat wave name *Zoe* via aided or unaided recall.

### Associations between messaging awareness and demographic variables

**Figure 3:**
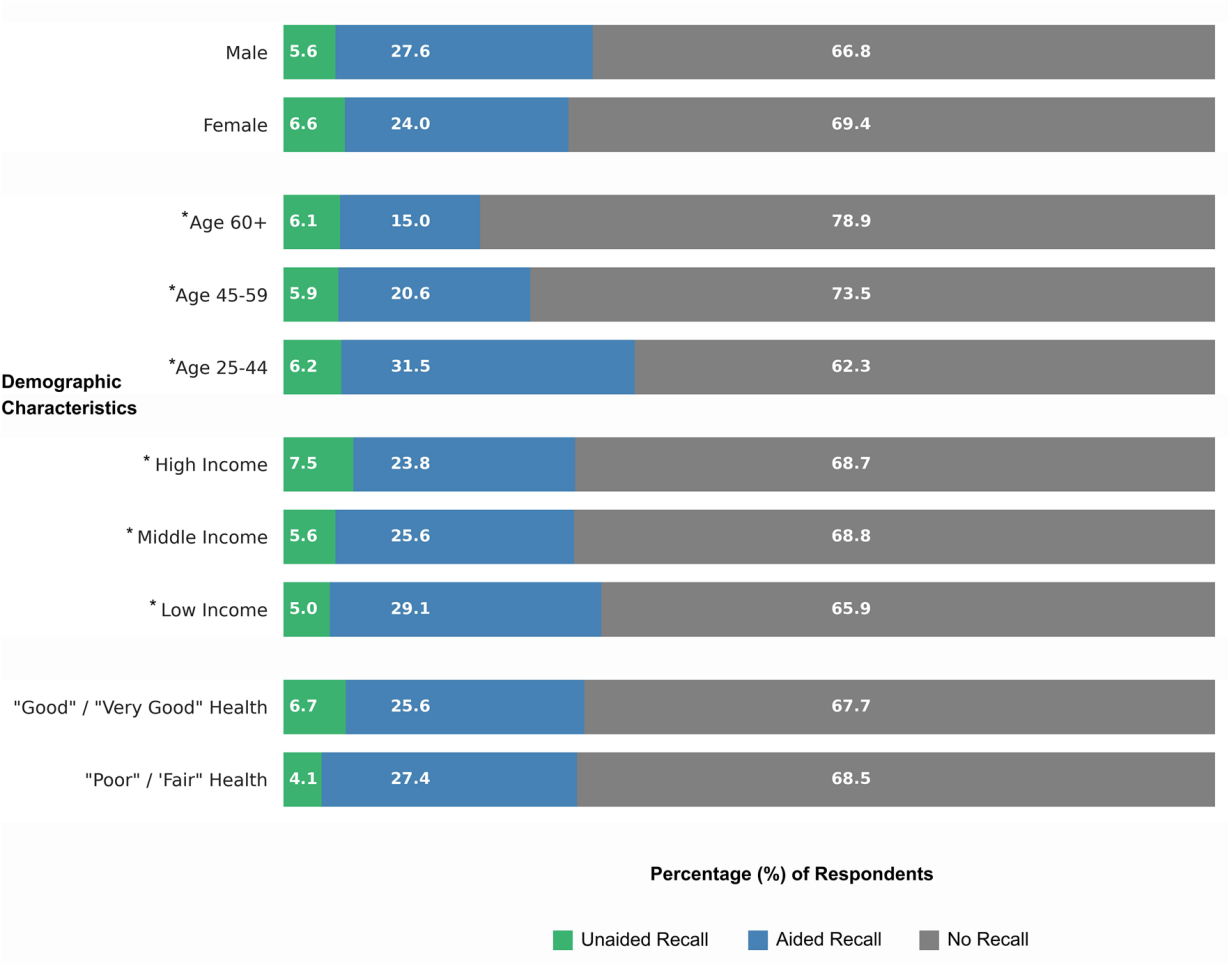
Bar charts depticing the percentage of respondents who reported unaided, aided, and no recall within various demographic categories.

Separate between factors ANOVA were run for age, income, and health status with awareness groups as the independent factor (no awareness, aided, unaided). Awareness groups significantly differed by age (F(2,2019) = 33.63, *p* < 0.001) and income (F(2,2019) = 5.21, *p* = 0.006) (Fig. 3). Participants in the no awareness group (M = 45.33, SD = 12.27) were, on average, older than both the unaided recall (M = 42.85, SD = 12.69) and aided recall (M = 40.30, SD = 11.22). Participants in the unaided recall groups (M = 6.97, SD = 2.61) reported higher monthly incomes than both the aided recall (M = 5.97, SD = 2.69) and no awareness (M = 6.21, SD = 2.58) groups. Awareness groups did not significantly differ by gender or health status.

### Associations between awareness of messaging and heat wave safety behaviors

**Figure 4:**
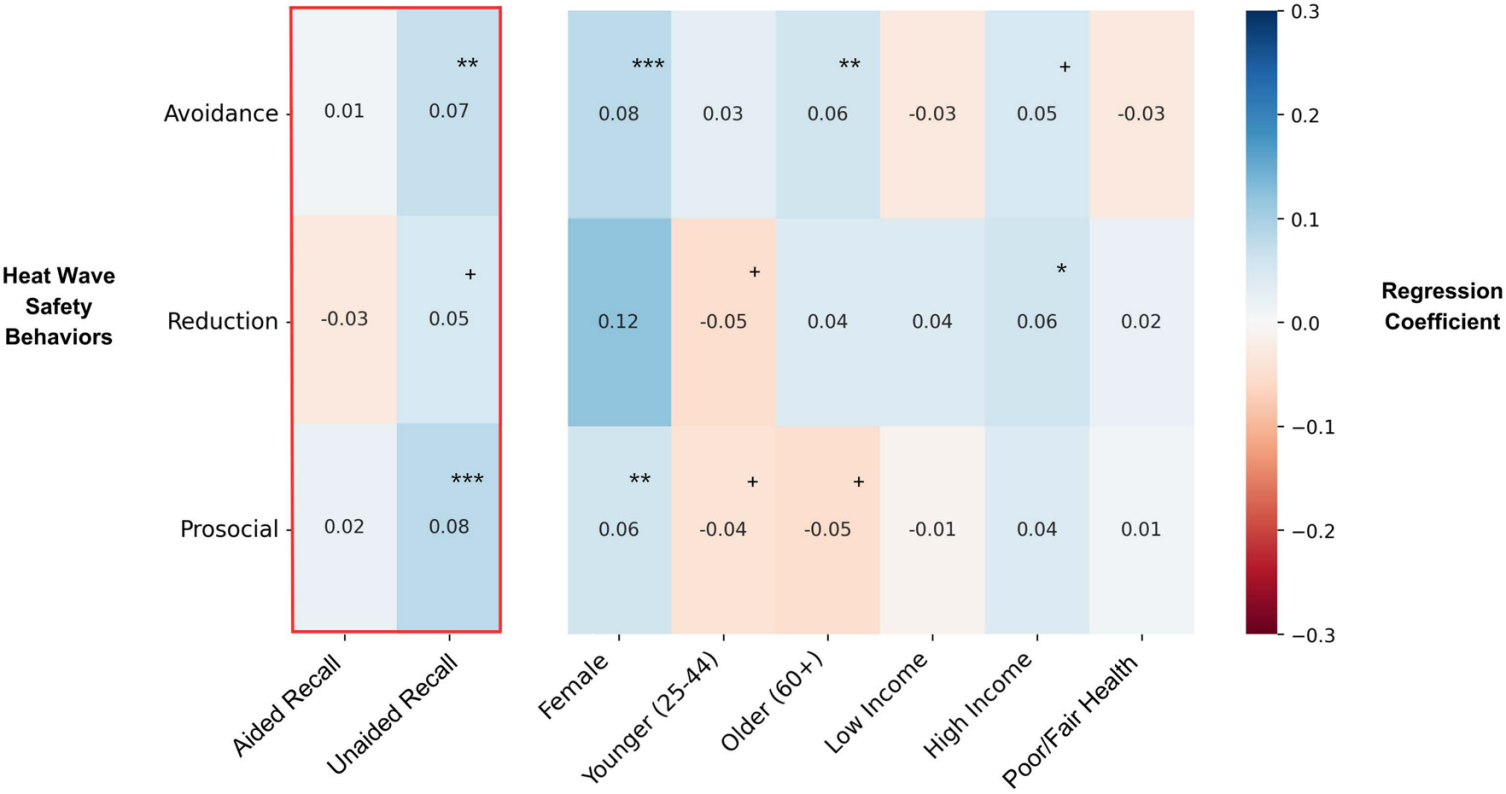
Heat map depticing the results of regression models predicting heat wave safety behaviors.

We fit a series of hierarchical regression models to examine associations between awareness of the messaging campaign and engagement in heat wave safety behaviors (Fig. 4). The dependent variables in these models were the total number of reported behaviors for each separate heat wave safety behavior category (avoidance, reduction, prosocial). In step 1, the number of heat wave safety behaviors was regressed onto the demographic variables (gender, age, income, health status). In step 2, unaided and aided awareness were added to the model. Full regression model results are included in the supplemental materials.

**Figure 5:**
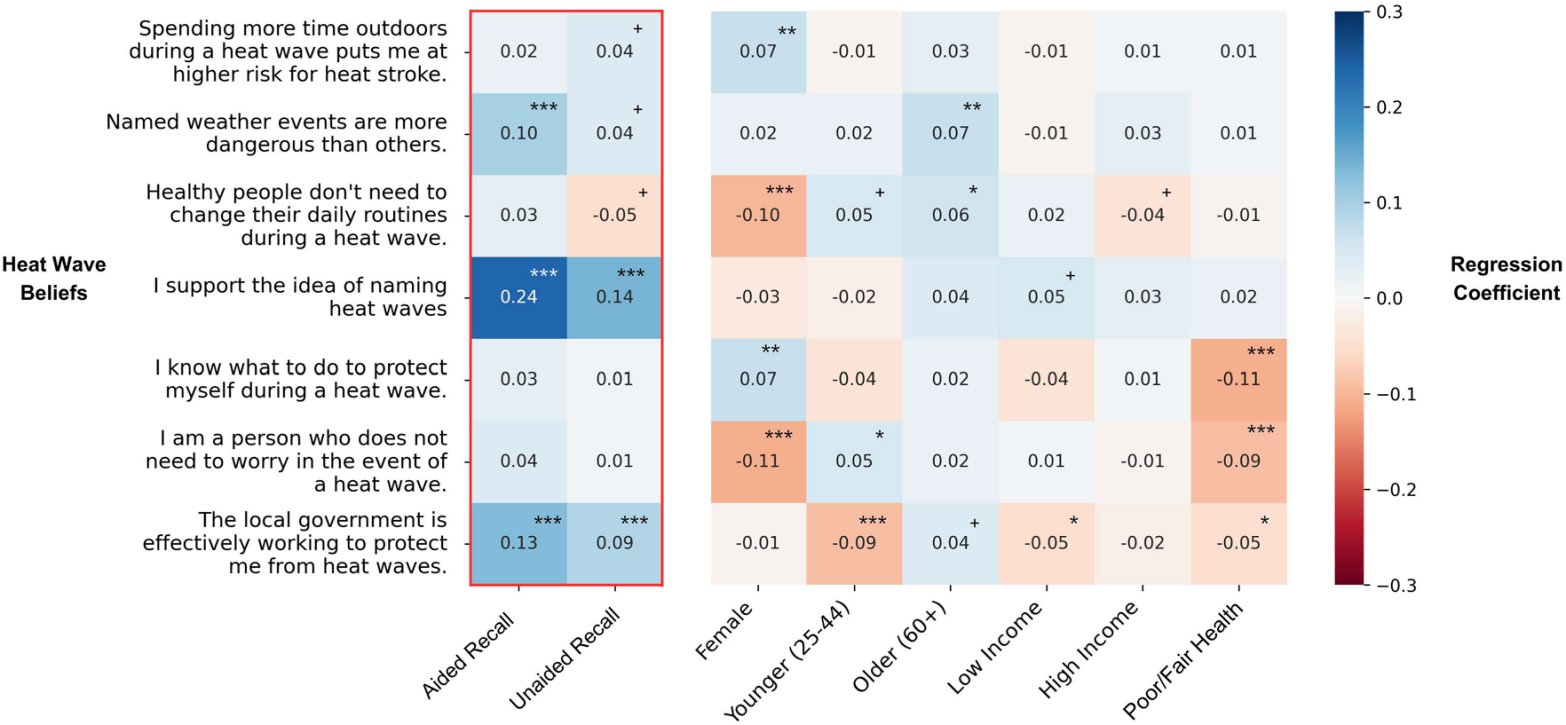
Heat map depicting the results of regression models predicting heat wave beliefs.

Several demographic variables emerged as statistically significant predictors of heat wave safety behavior. Women, older adults, and wealthier individuals engaged in more behaviors than men across all three behavior categories (see Fig. 5 and supplemental materials for a full discussion).

After adjusting for demographic correlates, unaided awareness was statistically significantly associated with avoidant (B = 0.27, SE = 0.09, *p* = 0*.*002) and prosocial behaviors (B = 0.26, SE = 0.07, *p* < 0*.*001) and marginally associated with reduction behaviors. Those who recalled the name *Zoe*, unaided, engaged in between 0.2 and 0.3 more heat wave safety behaviors than those who did not recall *Zoe*.

### Associations between messaging awareness and heat wave beliefs

We fit analogous regression models for each of the measures of heat wave perception and beliefs (Fig. 5). Women and older adults were more likely to agree with protective heat wave beliefs (e.g., spending time outdoors increases the risk of heat stroke, and that they knew how to stay safe during a heat wave). Men and younger adults were more likely to agree with what have been called “impervious” heat beliefs (e.g., that they are not the kind of person who needs to worry about heat waves, and that healthy people do not need to change their behavior)^19^. Older adults were more likely to agree that the local government is doing a good job of protecting its citizens, and lower income and poor health status respondents were less likely to agree. Poor health status respondents were also less likely to agree that they know what to do to stay safe during a heat wave and that they are not the kind of person who needs to worry about heat waves. See Fig. 5 and supplemental materials for a full discussion of demographic results.

Over and above these demographic factors, awareness of the named heat wave *Zoe* was associated with three heat wave beliefs. Compared to those with no awareness, participants with aided awareness of *Zoe* were more likely to agree that named weather events are more dangerous than other storms (that do not have names) (B = 0.27, SE = 0.08, *p* < 0.001; aided somewhat/strongly agree = 52.2%, no recall somewhat/strongly agree = 39.9%). Compared to those with no awareness of *Zoe*, both aided and unaided recall of *Zoe* were associated with greater support for the naming of heat waves (aided: B = 0.56, SE = 0.05, *p* < 0.001, unaided: B = 0.60, SE = 0.09, *p* < 0.001; aided strongly agree = 24.9% unaided recall strongly agree = 27.9%, no recall strongly agree = 9.5%). In addition, compared to those with no awareness of *Zoe,* both aided and unaided recall of *Zoe* was associated with agreement that the local government is working to protect its citizens and support for naming heat waves (aided: B = 0.34, SE = 0.06, *p* < 0.001, unaided: B = 0.43, SE = 0.11, *p* < 0.001; aided somewhat/strongly agree = 36.1% unaided recall strongly agree = 37.4%, no recall strongly agree = 23.3%).

## Discussion

Notwithstanding abundant evidence of the health threats posed by extreme heat and substantial investments in heat early warning and response systems, extreme heat events continue to be associated with large numbers of deaths each year. The high death toll posed by heat suggests a need for new approaches to communicate and reduce risk. The current study examined the impacts of a specific public health messaging strategy, naming heat events classified as the most severe in terms of potential impacts on excess mortality. Specifically, we sought to evaluate whether people residing in the Seville area during summer 2022 recalled heat wave *Zoe* as an indicator of their awareness of the associated heat wave messaging campaign. In a survey conducted at the end of the summer, we found that 6% of respondents were able to recall *Zoe* without assistance and another 26% reported recalling heat wave *Zoe* with prompting. Aided recall was most common among younger individuals and unaided recall was most common among individuals reporting higher monthly incomes.

Although at the time of this study, naming of heat waves was unprecedented, naming of other types of weather events is commonly used to communicate that event’s severity and potential impacts both internally and to the public^22^. For example, in the United States, hurricanes have received names since the early 1950s^23^. Naming extreme weather events is generally accepted by the public, including among those who have had previous negative experiences with weather events^24^. There is mixed empirical evidence for whether naming effectively conveys the severity of a weather event, influences preparation, or encourages behavior uptake to reduce health risks associated with the event. For instance, laboratory research has found that the inclusion of a storm name in storm descriptions has negligible impact on participants’ ratings of the storm’s severity^25,26^. In an experiment presenting mock Twitter posts from The Weather Channel about a winter storm, there was no significant difference in perception of storm severity between participants who were shown a post with a named storm and those shown a post without the naming component^25^. Another study found that severity perceptions were strongest in the no-name condition followed by named storm conditions^26^. Limited research suggests that the kind of name chosen for a storm (i.e. a humorous, uncommon, or common name, etc.) may affect risk perception^25,26^. Importantly, most of the previous research on naming weather events has been conducted in controlled, laboratory settings, and there is less research on perception of real-life named storms or whether awareness of a named storm affects behavior or attitudes.

 The limited available evidence suggests that commonly issued heat alerts (i.e., without names) are not having the intended effect of lowering risk of heat-related deaths^27,28^. In contrast to previous laboratory experiments, the current study was based on a real-life named heat wave. Potentially, because the extreme weather event was actually experienced by participants rather than presented as a hypothetical scenario, the salience of heat wave-related messages and awareness of the heat wave name may have been higher than in laboratory settings.

A number of behaviors may help individuals reduce the health risks associated with exposure to extreme heat^3,29^, and previous research suggests that beliefs about heat waves may affect engagement in heat wave safety behaviors^19,29,30^. Previous research has found that individuals engage in a wide range of behavioral strategies to cope with elevated heat, with varying efficacy^19^. Accordingly, we hypothesized that public messaging campaigns that convey the health risks of extreme heat and encourage heat wave safety behaviors could help reduce the negative health impacts of extreme heat events. The current study provides initial evidence for the potential utility of naming heat waves as part of a comprehensive heat health early warning system and communications campaign. Individuals who were aware of the messaging campaign and could recall the heat wave name *Zoe* reported engagement in statistically significantly more heat wave safety behaviors and also had significantly more favorable attitudes toward heat wave naming and their local government’s efforts to keep its citizens safe from heat.

Awareness of the heat wave messaging campaign differed based on several demographic characteristics. Participants who recalled the name *Zoe* tended to be younger and have higher incomes than those who were not aware of the campaign. Younger, wealthier people are more likely to utilize social media platforms^31^, which was a primary medium through which the heat wave naming campaign was distributed. In addition, individual demographic characteristics such as gender, age, health status, and socioeconomic status were also significant predictors of both engagement in heat wave safety behaviors and beliefs about heat waves. Thus, the proMETEO Sevilla messaging campaign may have disproportionately reached those that were already at lower risk of adverse health impacts and/or already engaging in more adaptive behaviors. It would be useful for future studies to examine how to best tailor messaging to reach those individuals or communities thought to be at greatest risk or least engaged in personal adaptive behaviors.

Although further research is needed, our initial assessment has potential implications for public health policy. Given the health risks associated with extreme heat, encouraging engagement in heat wave safety behavior and bolstering community emergency response is a reasonable and common priority for local, regional, national, and international health institutions^14^. The current study indicates that naming a heat wave is associated with increased engagement in a range of protective behaviors, including avoidance, reduction, and prosocial behaviors. Interestingly, awareness of *Zoe* was associated with greater engagement in prosocial activities, particularly communicating with others and warning others about heat waves. Prosocial activities may be especially important, as they are least engaged in during heat waves^19^, yet may serve as an important mechanism for increasing community resilience and reaching the most vulnerable members of a community^32,33^. Naming heat waves may help to facilitate communication among community members by providing a common identifier, which may make the extreme heat event more tangible and easy to talk about.

While awareness of *Zoe* was not associated with each individual measure of beliefs, the beliefs that were associated with awareness of *Zoe* provide important insights for public health policy and public health messaging efforts. Findings provide initial evidence that exposure to heat wave names may increase individuals’ perception of the dangers of heat waves, as those who remembered the name *Zoe* via aided recall more strongly agreed that named weather events are more dangerous than other weather events.

While *Zoe* was the first named heat wave, by the end of August 2023 an additional 3 named heat waves had been named through proMETEO Sevilla (Yago, June 25–27; Xenia, July 10–July 13; and Wenceslao, August 7–13). Following the summer of 2023, there is additional opportunity for future research to evaluate the utility of naming heat waves, and under which conditions naming and associated messaging is well received by communities and effective at reducing heat health risks. *Zoe* was named with and through partnerships with the City of Seville and the University of Seville, which may have contributed to the finding that those who recalled *Zoe* had greater trust that their local government is working to protect them from heat. The experience in Seville suggests that public trust in the organization leading the naming and communication is essential to reaching and impacting a wide audience, although we were not able to directly test this hypothesis in this study.

### Limitations and future research

The current study’s findings need to be interpreted in light of its methodological limitations. Our analyses are based on self-reported data, which is prone to measurement error including social desirability biases. Although the survey was administered in the fall following the heat wave (1–1.5 months later), self-report measures are also susceptible to respondent recall errors. In addition, the studied regions in Spain continue to experience warm temperatures during the months of September and early October (when the survey was fielded), so participants’ responses may have been influenced by the warm weather they were currently experiencing. However, all survey questions primed respondents to consider their behaviors and attitudes during the most recent extreme heat event they had experienced. In addition, the current study measured eleven key heat wave safety behaviors that were representative of the three behavior categories, but future research should consider measuring additional behaviors such as additional personal cooling strategies (use of fans, air conditioning, etc.) The primary independent variable was recall of the heat wave name *Zoe* and overall recall levels (both aided and unaided) were fairly low in absolute terms. In addition, primary findings are based on correlational data. Although findings were derived from models controlling for a number of demographic and socioeconomic factors, it is not possible to rule out alternative explanations for the found associations. Future research using longitudinal data to track individual change in behavior/attitudes over time in relation to message campaign awareness could confirm or refute these findings. In addition, there was not a comparison sample exposed to a similar heat wave messaging campaign without the name *Zoe*. In future research, experimental designs could be used to explore the impact of heat wave messaging campaigns that include the naming component vs. those that do not include a heat wave name. The overall reach of the proMETEO heat health naming and categorizing pilot messaging campaign was fairly limited. A retrospective study of the discussion activity across news networks and social networking sites, showed only a total of 140 posts mentioning both Zoe and the word heat wave (in English or Spanish), resulting in 37,500 impressions reaching about 6 million people (see supplemental materials). Future research on larger-scale pilots and messaging campaigns is warranted. Compared to the greater Spanish population, older adults were underrepresented in the current study sample, though older adults tend to be underrepresented in online research, in general, due to generational and technological barriers^21^.

It will also be important for future research to explore causal connections and potential mechanisms by which exposure to heat wave naming may be associated with increased engagement in heat wave safety behavior. For instance, seeing that a heat wave has been named may increase beliefs that the event is serious and worthy of an individual’s attention, which, in turn, could lead individuals to take more precautions. Naming a heat wave could also ease communication and dissemination of information about the heat wave both through formal channels, such as news outlets, and informal channels, such as discussions among individuals in person and on social media. In addition, while the current study statistically controlled for risk factors such as age and health status, future research should explore whether heat wave naming campaigns differentially affect the heat wave safety behaviors of vulnerable groups (i.e., moderation analysis) such as older adults, individuals with pervasive health issues, pregnant women, etc. Finally, the messaging campaign around *Zoe* was part of a HHEWS categorizing and naming pilot project, but recall of the specific categorizing system was not related to engagement in heat wave safety behavior or beliefs toward heat waves. Future research could include additional measures which further assess individual’s understanding of the links between the heat wave name and heat health early warning systems or categories.

## Conclusions

Heat waves pose an unambiguous public health risk, and the frequency, intensity, and duration of heat waves will increase in the coming years due to climate change^33^.

The current study provides initial evidence that naming a heat wave as part of a comprehensive HHEWS may encourage individuals to engage in more heat wave safety behaviors.This study also found a positive association between naming a heat wave and trust in local government response to heat waves. Although naming of heat wave Zoe was associated with heightened awareness and engagement in protective behaviors and beliefs, it is unclear whether these impacts are causally linked or whether similar impacts would be observed in other locations or other points in time as the novelty of named heat waves declines. This preliminary research suggests that naming heat waves may increase the perceived risk of extreme heat and increase residents’ perceptions that their local government is responding effectively to heat events. Naming may also encourage residents to translate that increased awareness into action by doing more to protect themselves and their communities from extreme heat. Naming interventions should continue to be tested and expanded to evaluate if these preliminary benefits of increased community awareness and action are reproducible and consistent. Naming heat waves represents a novel, potentially useful approach to raising awareness of heat health dangers and encouraging protective behaviors among individuals. However, more evidence of its potential benefits (and drawbacks or concerns) is needed before any policy recommendations can be made.

### Supplementary Information

Supplementary Information 1.

Supplementary Information 2.

## Data Availability

All data generated or analyzed during this study are included in this published article in the supplemental files.
