# Supplementary material for: Beliefs and behaviors associated with the first named heat wave in Seville Spain 2022
Source: Sci Rep. 2024 Apr 20;14:9055. doi: 10.1038/s41598-024-59430-8 (PMC11032320; doi:10.1038/s41598-024-59430-8)
Supplement: Supplementary file 2 — Supplementary Information 2. [file 41598_2024_59430_MOESM2_ESM.docx]

**Additional Results**

*Demographic behaviors results:*

Compared to men, women engaged in more behaviors across all three categories (B’s = 0.10 - 0.19, SE’s = 0.04, *p*’s *<* 0*.*001); i.e. on average women engaged in 0.10 to 0.19 more heat wave safety behaviors than men. Compared to younger age groups, older adults engaged in 0.18 more avoidant behaviors, on average (B = 0.18, SE = 0.06, *p =* 0*.*009), while wealthier individuals engaged in 0.11 more heat wave reduction behaviors (B = 0.11, SE = 0.06, *p =* 0.016).

*Demographic beliefs results:*

***Gender differences:*** Gender was significantly associated with multiple heat wave beliefs. Women were more likely to agree that spending time outdoors increases the risk of heat stroke (B = 0.12, SE = 0.04, *p =* 0*.*004; women strongly agree = 72.8%, men strongly agree = 63.9%), and women also more strongly agreed that they knew how to stay safe during a heat wave (B = 0.11, SE = 0.04, *p =* 0*.*005; women strongly agree = 49.8%, men strongly agree = 45.0%). In contrast, men were more likely to agree that healthy people do not need to change their behavior (B = -0.23, SE = 0.05, *p <* 0*.*001; women somewhat/strongly agree = 11.4%, men somewhat/strongly agree = 17.8%) and that they are not the kind of person who needs to worry about heat waves (B = -0.26, SE = 0.05, *p <* 0*.*001; women somewhat/strongly agree = 16.4%, men somewhat/strongly agree = 24.3%).

***Age differences:*** Older adults (aged 60 and older) were more likely than younger adults to agree that named weather events are more dangerous (B = 0.25, SE = 0.09, *p =* 0*.*004; older adults strongly agree = 21.5%, younger adults strongly agree = 17.0%) and healthy people don’t need to change their routines during heat waves (B = 0.22, SE = 0.09, *p =* 0*.*011; older adults strongly agree = 22.7%, younger adults strongly agree = 14.3%). Younger adults were more likely to agree than older adults that they are a person who does not need to worry about heat waves (B = 0.12, SE = 0.06, *p =* 0*.*037; younger adults strongly *disagree* = 26.9%, older adults strongly *disagree* = 34.2%), but younger adults were *less* likely to agree that the local government is doing a good job of protecting its citizens than older adults (B = -0.20, SE = 0.06, *p <* 0*.*001; older adults somewhat/strongly agree = 30.8%, younger adults strongly agree = 24.8%).

***Income differences:*** Compared to individuals with higher monthly incomes, individuals with lower monthly incomes (1300 euros per month or less) were less likely to agree that the local government is doing a good job of protecting its citizens from heat (B = -0.13, SE = 0.06, *p =* 0*.*042; lower income strongly *disagree* = 21.2%, higher income strongly agree = 15.4%).

***Health status differences:*** Compared to those who self-reported better health, those who self-reported poorer health over the past 12 months were less likely to agree that they know what to do to stay safe during a heat wave (B = -0.19, SE = 0.04, *p <* 0.001; poorer health strongly agree = 41.6%, better health strongly agree = 49.5%), that they are not the kind of person who needs to worry about heat waves (B = -0.19, SE = 0.04, *p <* 0.001; poorer health strongly agree = 41.6%, better health strongly agree = 49.5%), and that the local government is doing a good job of protecting its citizens from the dangers of heat (B = -0.19, SE = 0.04, *p <* 0.001; poorer health strongly agree = 41.6%, better health strongly agree = 49.5%).

Table 1. *Characteristics of study sample in comparison to population of Spain.*

|  | Sample | Population of Spain |
| --- | --- | --- |
| GENDER  Female | 54.3% | 50.8% |
| AGE |  |  |
| 25-34 | 26.5% | 15.5% |
| 35-44 | 30.2% | 20.7% |
| 45-54 | 19.2% | 21.8% |
| 55-64 | 18.0% | 18.2% |
| 65-74 | 5.3% | 12.6% |
| 75+ | 0.8% | 11.2% |

Table 2. *Frequencies of heat wave safety behaviors (n = 2022)*

|  | *n* | *%* |
| --- | --- | --- |
| Avoidance Behaviors |  |  |
| Spent more time inside | 1174 | 58.1% |
| Changed leisure plans | 790 | 39.1% |
| Changed work hours | 154 | 7.6% |
| Worked from home | 133 | 6.6% |
| Found place to cool off  Engaged in any avoidant behavior | 243  1557 | 12.0%  77.0% |
| Reduction Behaviors |  |  |
| Drank more water | 1291 | 63.8% |
| Dressed differently | 346 | 17.1% |
| Changed what I ate  Engaged in any reduction behavior | 342  1397 | 16.9%  69.1% |
| Prosocial Behaviors |  |  |
| Warned others | 565 | 27.9% |
| Talked about ways to stay safe | 326 | 16.1% |
| Helped others  Engaged in any prosocial behavior | 167  750 | 8.3%  37.1% |

Table 3. *Descriptive statistics for heat wave perceptions and attitudes measures (n = 2022)*

|  | *Mean* | *SD* | *Range* | *Partially / Completely agree* |
| --- | --- | --- | --- | --- |
| Perceptions about Heat Waves |  |  |  |  |
| 1. Spending more time outdoors during a heat wave puts me at higher risk for heat stroke. | 4.52 | .88 | 1 - 5 | 89.1% |
| 1. Named weather events are more dangerous than others | 3.30 | 1.18 | 1 - 5 | 42.6% |
| 1. Healthy people don't need to change their daily routines during a heat wave. | 2.03 | 1.17 | 1 - 5 | 15.4% |
| 1. I support the idea of naming heat waves | 3.24 | 1.02 | 1 - 5 | 29.3% |
| Self-efficacy beliefs about heat waves |  |  |  |  |
| 1. I know what to do to protect myself during a heat wave. | 4.34 | .77 | 1 - 5 | 89.4% |
| 1. I am a person who does not need to worry in the event of a heat wave. | 2.33 | 1.19 | 1 - 5 | 20.0% |
| Government responsibility to protect citizens |  |  |  |  |
| 1. The local government is effectively working to protect me from heat waves | 2.78 | 1.15 | 1 - 5 | 27.5% |

NOTE: 1 = Completely disagree, 2 = Disagree, 3 = Neither agree nor disagree, 4 = Agree, 5 = Completely agree

Table 4. *Regression models predicting heat wave safety behaviors*

|  | Avoidance | | | | Reduction | | | | Prosocial | | | |
| --- | --- | --- | --- | --- | --- | --- | --- | --- | --- | --- | --- | --- |
| STEP 1 | B | SE | 𝛃 | p | B | SE | 𝛃 | p | B | SE | 𝛃 | p |
| Gender (Female) | .15 | .04 | .08*** | <.001 | .19 | .04 | .12*** | <.001 | .10 | .04 | .06** | .007 |
| Age: younger (25-44)^+^ | .05 | .05 | .03 | .288 | -.08 | .04 | -.05+ | .053 | -.07 | .04 | -.04+ | .086 |
| Age: older (60+)^+^ | .18 | .07 | .06** | .009 | .09 | .06 | .04 | .137 | -.11 | .06 | -.05+ | .056 |
| Monthly Income: low^#^ | -.06 | .05 | -.03 | .212 | .07 | .05 | .04 | .157 | -.01 | .04 | -.01 | .833 |
| Monthly Income: high^##^ | .09 | .05 | .05+ | .062 | .11 | .05 | .06* | .016 | .07 | .04 | .04 | .110 |
| Health status: poor/fair | -.07 | .05 | -.03 | .151 | .03 | .04 | .02 | .652 | .02 | .04 | .01 | .583 |
| STEP 2 |  |  |  |  |  |  |  |  |  |  |  |  |
| Aided awareness | .01 | .05 | .01 | .879 | -.05 | .04 | -.03 | .215 | .04 | .04 | .02 | .329 |
| Unaided awareness | .27 | .09 | .07** | .002 | .13 | .06 | .05+ | .088 | .26 | .07 | .08*** | <.001 |
| R^2^ | .02 | | | | .02 | | | | .01 | | | |

NOTE: ^+^ Middle-aged adults (45-59) are the comparison group. ^#^ Income = Under 1300 euros per month, ^##^ Income = 2200 euros per month or more. + *= p* < .1, **p* < .05, ***p* < .01, ****p* < .001

Table 5a. *Regression models predicting heat wave perceptions*

|  | Spending more time outdoors during a heat wave puts me at higher risk for heat stroke. | | | | Named weather events are more dangerous than others | | | | Healthy people don't need to change their daily routines during a heat wave. | | | | I support the idea of naming heat waves | | | |
| --- | --- | --- | --- | --- | --- | --- | --- | --- | --- | --- | --- | --- | --- | --- | --- | --- |
| STEP 1 | B | SE | 𝛃 | p | B | SE | 𝛃 | p | B | SE | 𝛃 | p | B | SE | 𝛃 | p |
| Gender (Female) | .12 | .04 | .07** | .004 | .02 | .06 | .02 | .397 | -.23 | .05 | -.10*** | <.001 | -.05 | .05 | -.03 | .227 |
| Younger (25-44)^+^ | -.01 | .04 | -.01 | .881 | .05 | .06 | .02 | .397 | .11 | .06 | .05+ | .063 | -.03 | .05 | -.02 | .496 |
| Older (60+)^+^ | .07 | .07 | .03 | .295 | .25 | .09 | .07** | .004 | .22 | .09 | .06* | .011 | .11 | .07 | .04 | .124 |
| Low Income^#^ | -.01 | .05 | -.,01 | .976 | -.01 | .07 | -.01 | .873 | .04 | .07 | .02 | .565 | .10 | 06 | .05+ | .065 |
| High Income^##^ | .02 | .05 | .01 | .704 | .06 | .06 | .03 | .333 | -.10 | .06 | -.04+ | .098 | .07 | .05 | .03 | .185 |
| Poor/fair  Health | .02 | .05 | .01 | .671 | .02 | .06 | .01 | .780 | -.03 | .06 | -.01 | .600 | .04 | .05 | .02 | .413 |
| STEP 2 |  |  |  |  |  |  |  |  |  |  |  |  |  |  |  |  |
| Aided awareness | .04 | .05 | .02 | .403 | .27 | .08 | .10*** | <.001 | .07 | .06 | .03 | .221 | .56 | .05 | .24*** | <.001 |
| Unaided awareness | .15 | .08 | .04+ | .069 | .13 | .08 | .04+ | .086 | -.22 | .11 | -.05+ | .057 | .60 | .09 | .14*** | <.001 |
| R^2^ |  |  | .007 | | .01 | | | | .02 | | | |  |  | .07 | |

+ *= p* < .1, **p* < .05, ***p* < .01, ****p* < .001

Table 5b. *Regression models predicting heat wave self-efficacy beliefs and attitudes toward local government*

|  | I know what to do to protect myself during a heat wave. | | | | I am a person who does not need to worry in the event of a heat wave. | | | | The local government is effectively working to protect me from heat waves | | | |
| --- | --- | --- | --- | --- | --- | --- | --- | --- | --- | --- | --- | --- |
| STEP 1 | B | SE | 𝛃 | p | B | SE | 𝛃 | p | B | SE | 𝛃 | p |
| Gender (Female) | .11 | .04 | .07** | .005 | -.26 | .05 | -.11*** | <.001 | -.02 | .06 | -.01 | .645 |
| Younger (25-44)^+^ | -.07 | .04 | -.04 | .081 | .12 | .06 | .05* | .037 | -.20 | .06 | -.09*** | <.001 |
| Older (60+)^+^ | .06 | .06 | .02 | .974 | .07 | .09 | .02 | .443 | .15 | .09 | .04 | .089+ |
| Low Income^#^ | -.06 | .04 | -.04 | .139 | .02 | .07 | .01 | .763 | -.13 | .06 | -.05* | 042 |
| High Income^##^ | .02 | .04 | .01 | .579 | -.01 | .06 | -.01 | .899 | -.05 | .06 | -.02 | .407 |
| Poor/fair  Health | -.19 | .04 | -.11*** | <.001 | -.26 | .06 | -.09*** | <.001 | -.14 | .06 | -.05* | .019 |
| STEP 2 |  |  |  |  |  |  |  |  |  |  |  |  |
| Aided awareness | -.06 | .04 | .03 | .154 | .10 | .06 | .04 | .12 | .34 | .06 | .13*** | <.001 |
| Unaided awareness | .04 | .07 | .01 | .615 | .06 | .11 | .01 | .597 | .43 | .11 | .09*** | <.001 |
| R^2^ |  |  | .02 | |  |  | .03 | |  |  | .03 | |

+ *= p* < .1, **p* < .05, ***p* < .01, ****p* < .001

Figure 1. *Age differences across awareness groups*


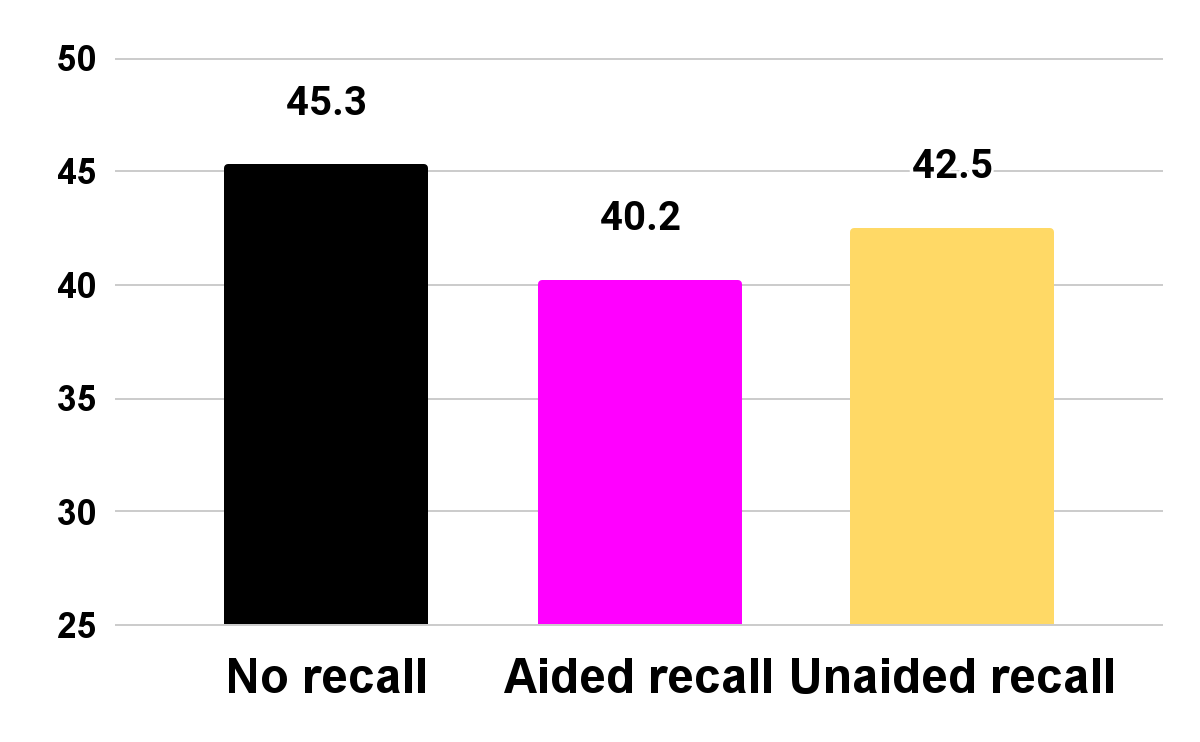


Figure 2. *Income differences across awareness groups*


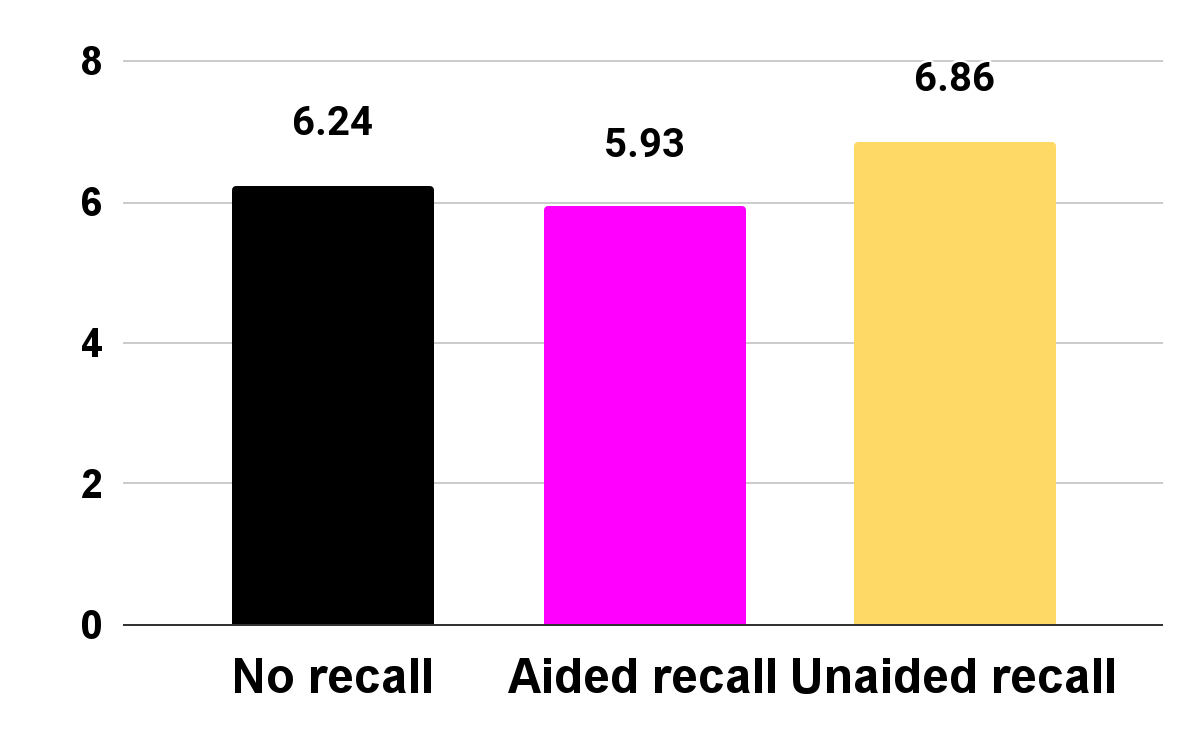


NOTE: Income scale: 4 = 1050 - 1300 euros per month, 5 = 1300 - 1550 euros per month, 6 = 1550 - 1800 euros per month


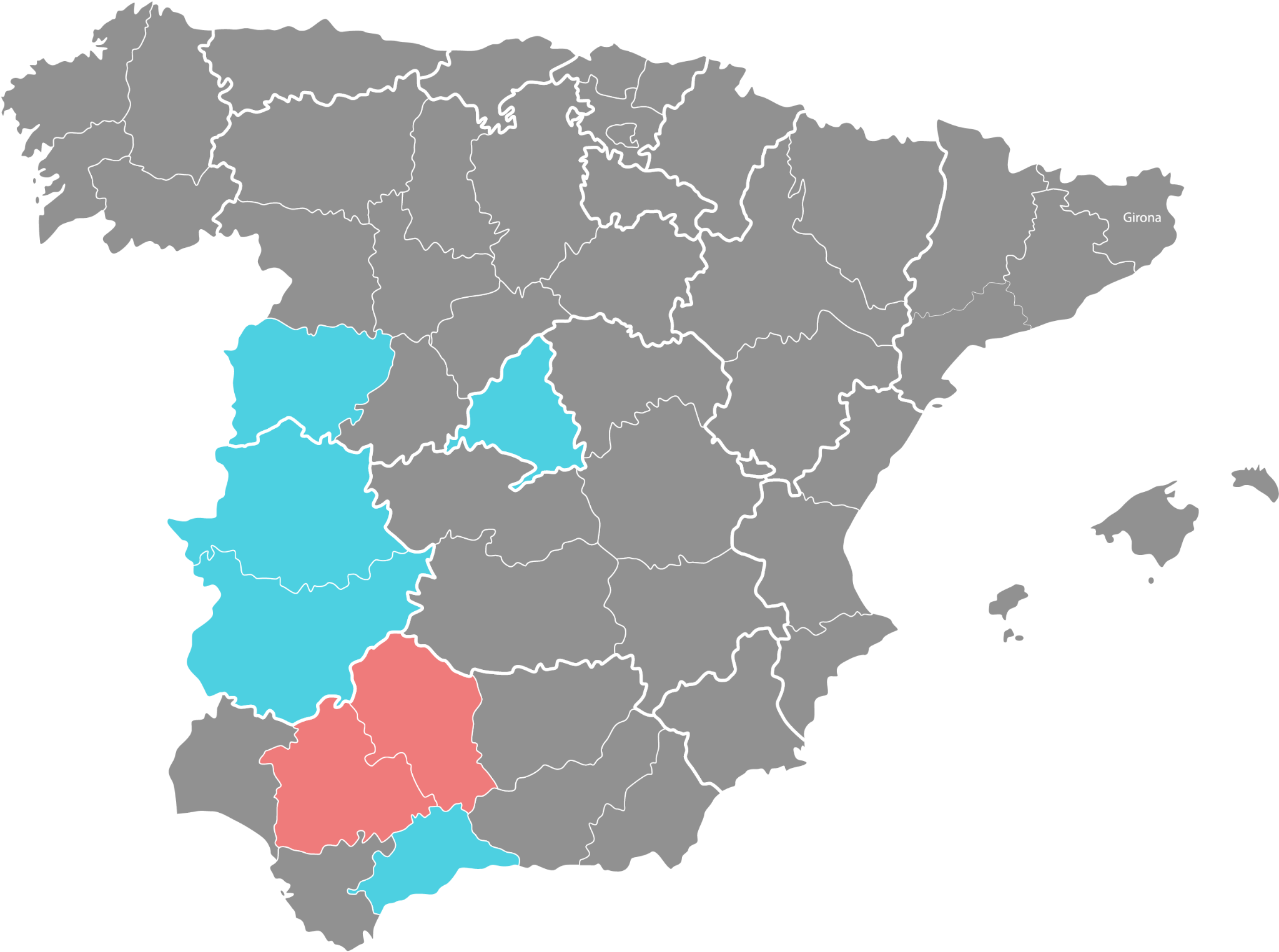


| **Intervention Regions** | **Control Regions** |
| --- | --- |
| **Córdoba**  **Seville** | **Badajoz**  **Cáceres**  **Madrid**  **Málaga**  **Salamanca** |

**proMETEO Messaging Campaign posts**

The proMETEO Sevilla campaign included social media notices and promotion of local and regional news coverage through local partners' social media channels. Contents of the messaging included:

- Tips on how to stay safe during heat waves, including heat wave Zoe
- Branding and visual identity for the system
- Heat waves and climate change
- Explanation of how heat wave naming and categorization work
- Posts also featured thermometer graphics to alert the public about the potential impacts of that day’s weather conditions.

The coverage of the campaign resulted in almost 5,000 impressions on the campaign social media channels across multiple platforms, including Facebook, Instagram, Twitter, and LinkedIn. Partner social posts promoting the project resulted in over 150 interactions.

The campaign also had its own website, prometeosevilla.com. The website had 550 return visitors and 543 new visitors who logged 2500 sessions averaging about one and a half minutes. The majority of website visits came from direct traffic and organic searches.

- [**Thermometer post**](https://twitter.com/PrometeoSevilla/status/1649472744868683791)

**
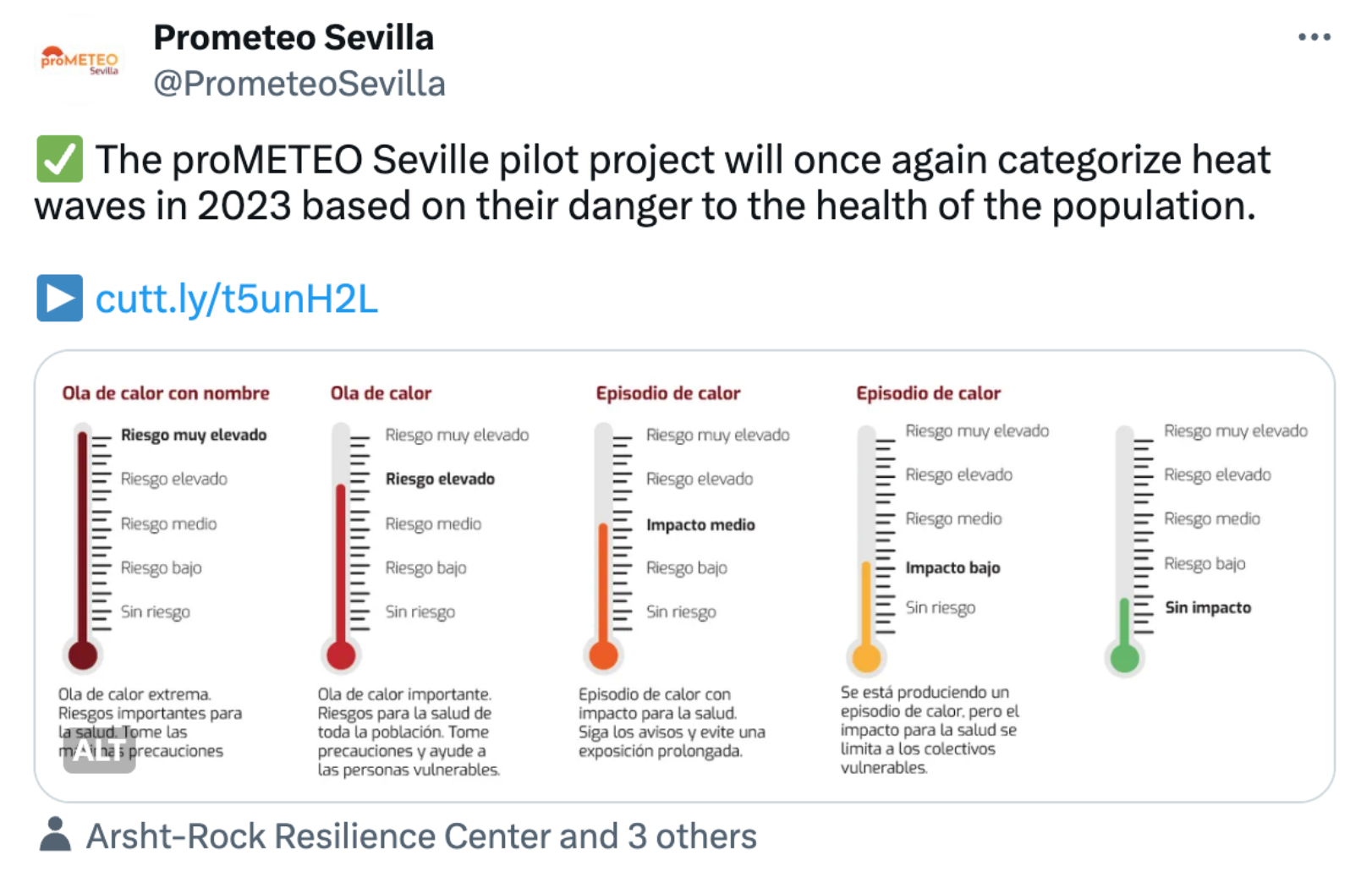
**

**
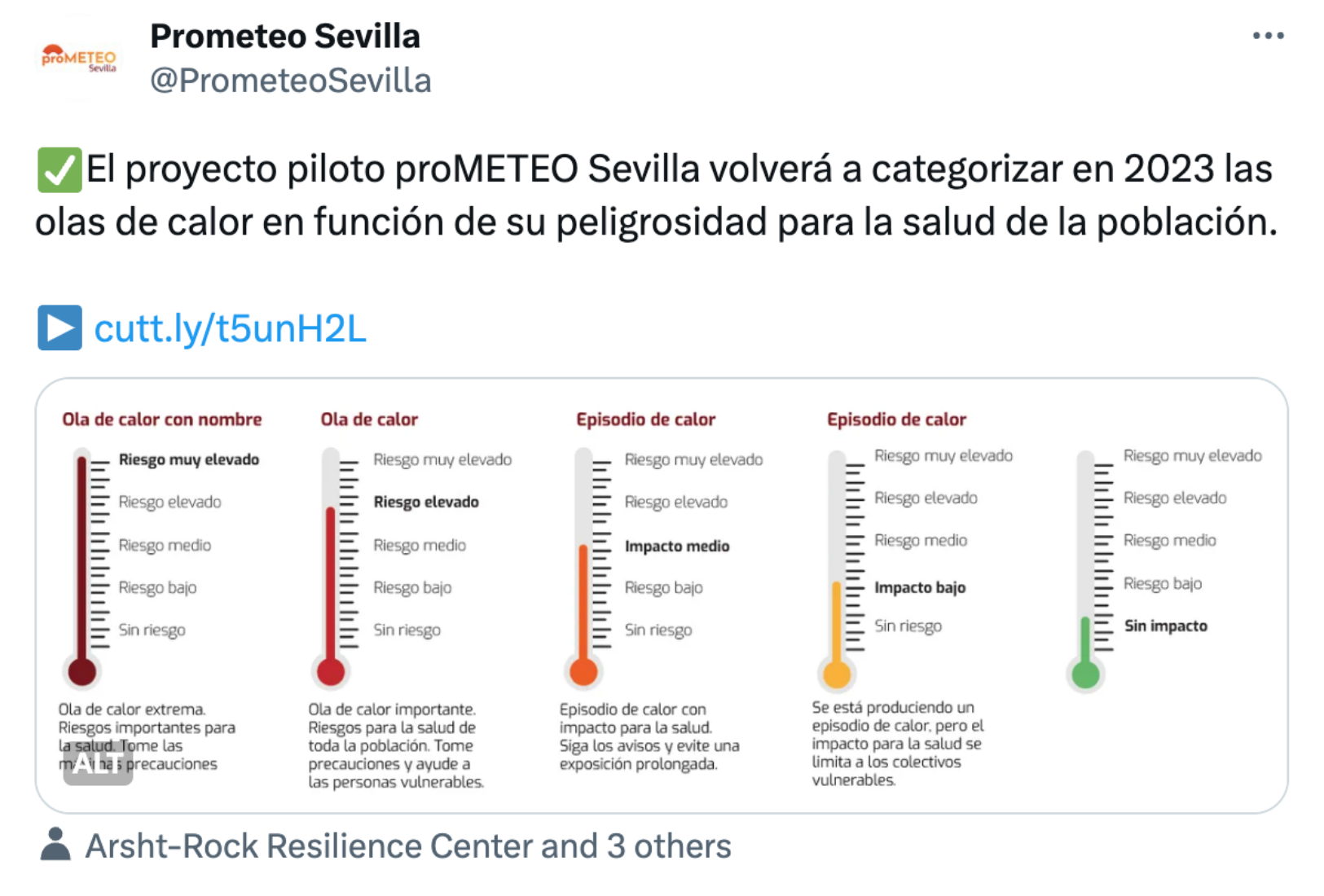
**

- [**Initial Zoe announcement**](https://twitter.com/PrometeoSevilla/status/1551472455553880066)

**
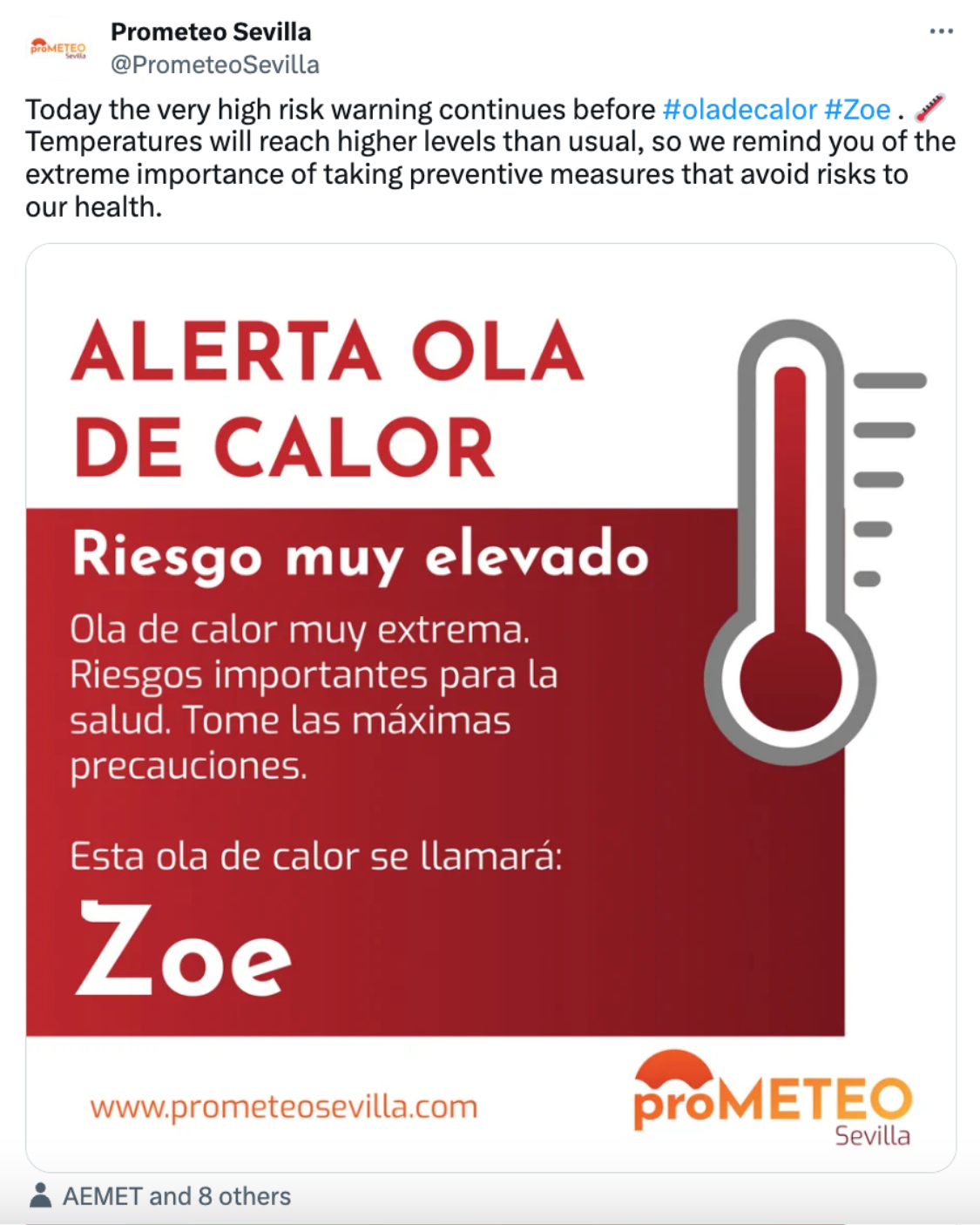
**

**
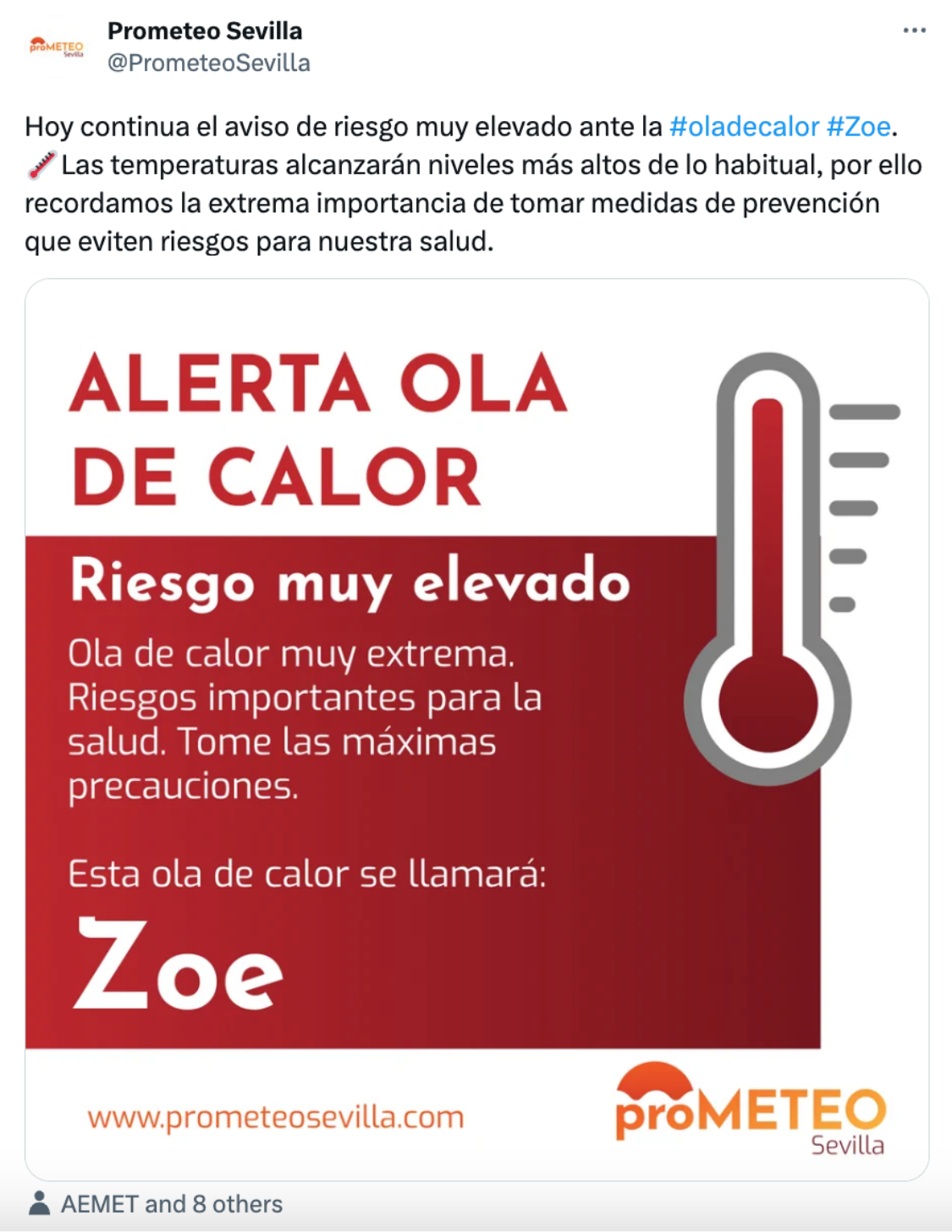
**

- [**Health tips shared during Zoe**](https://twitter.com/PrometeoSevilla/status/1551526998400548867)


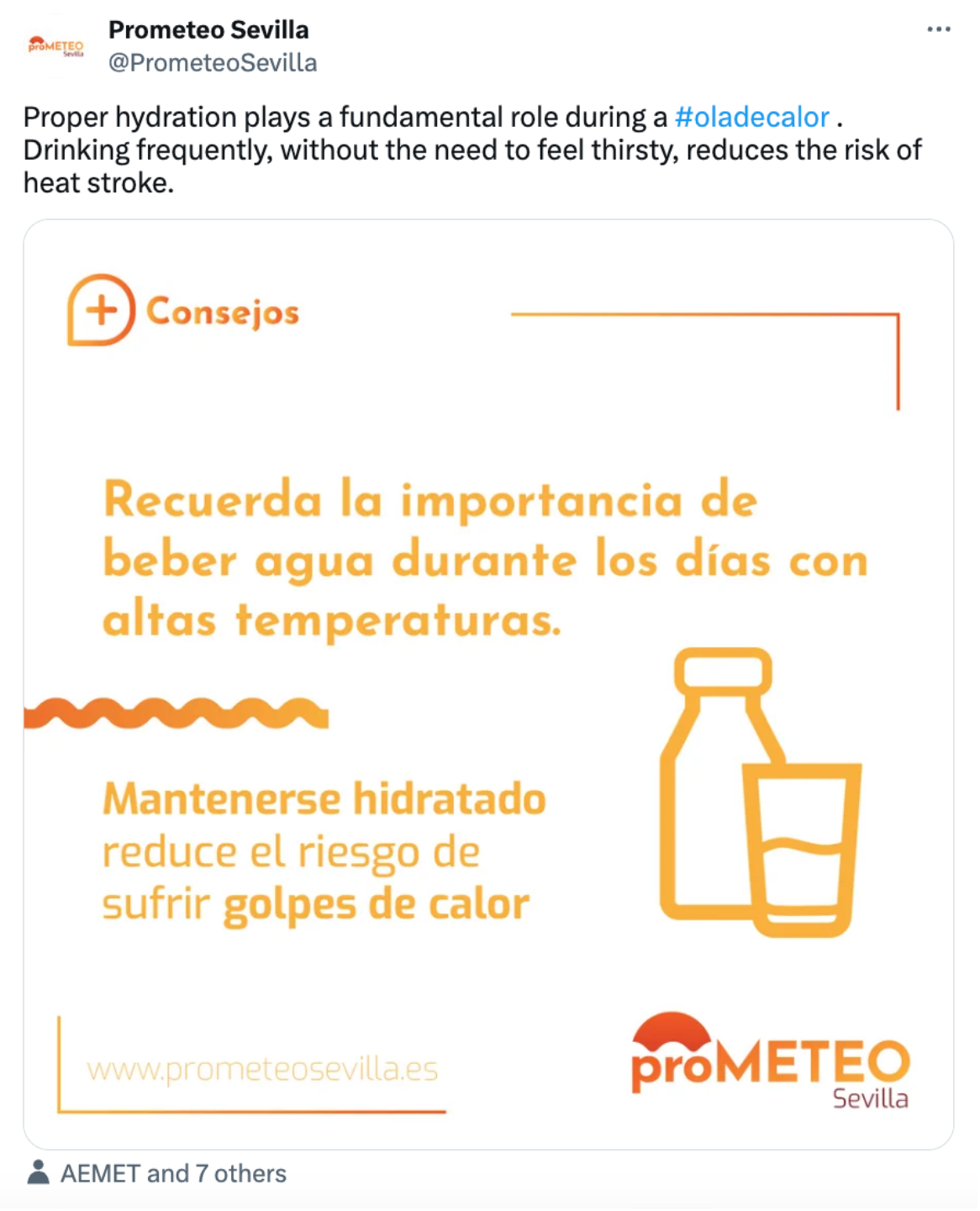


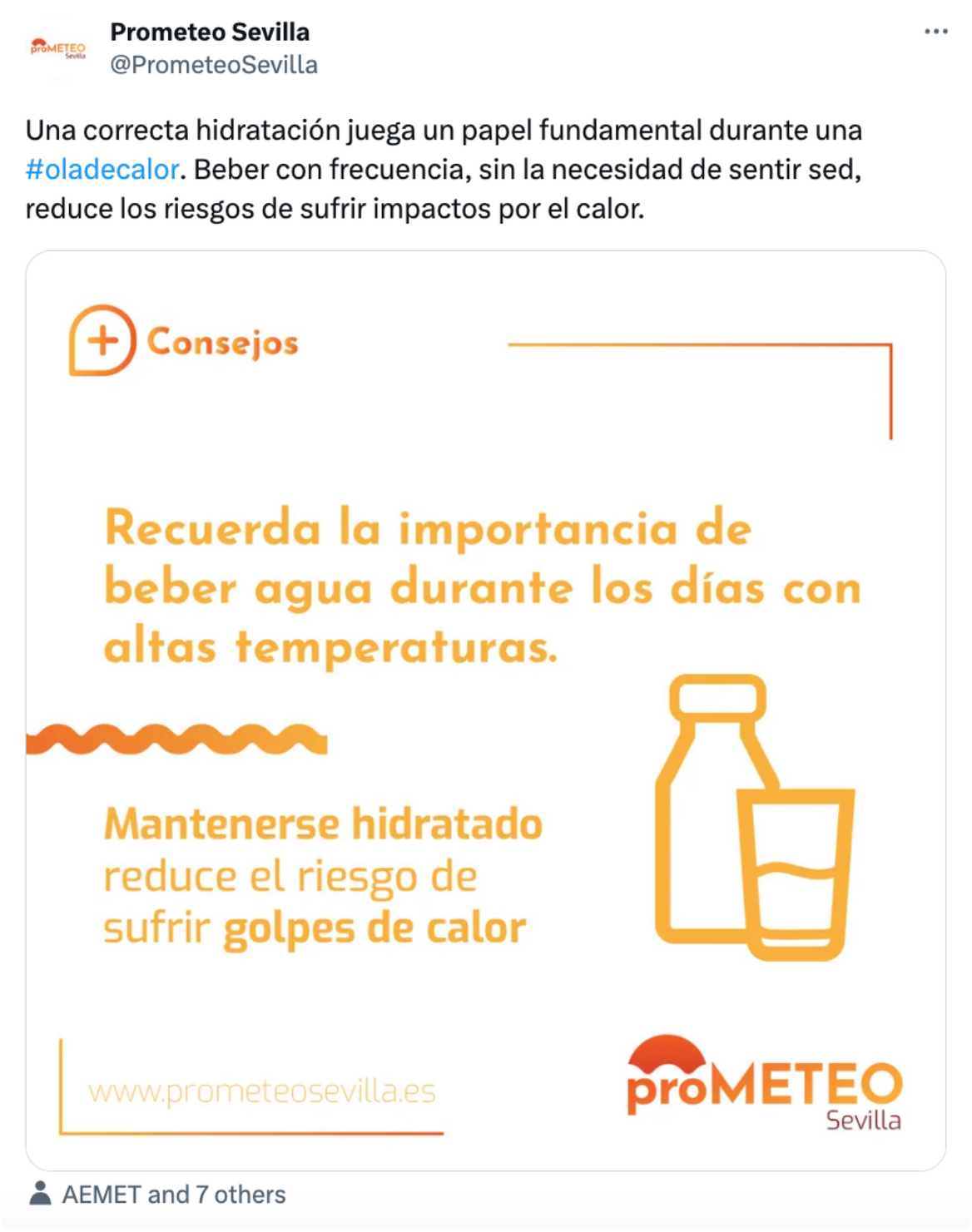


- [**Health tips part 2**](https://twitter.com/PrometeoSevilla/status/1551575500446871552)

**
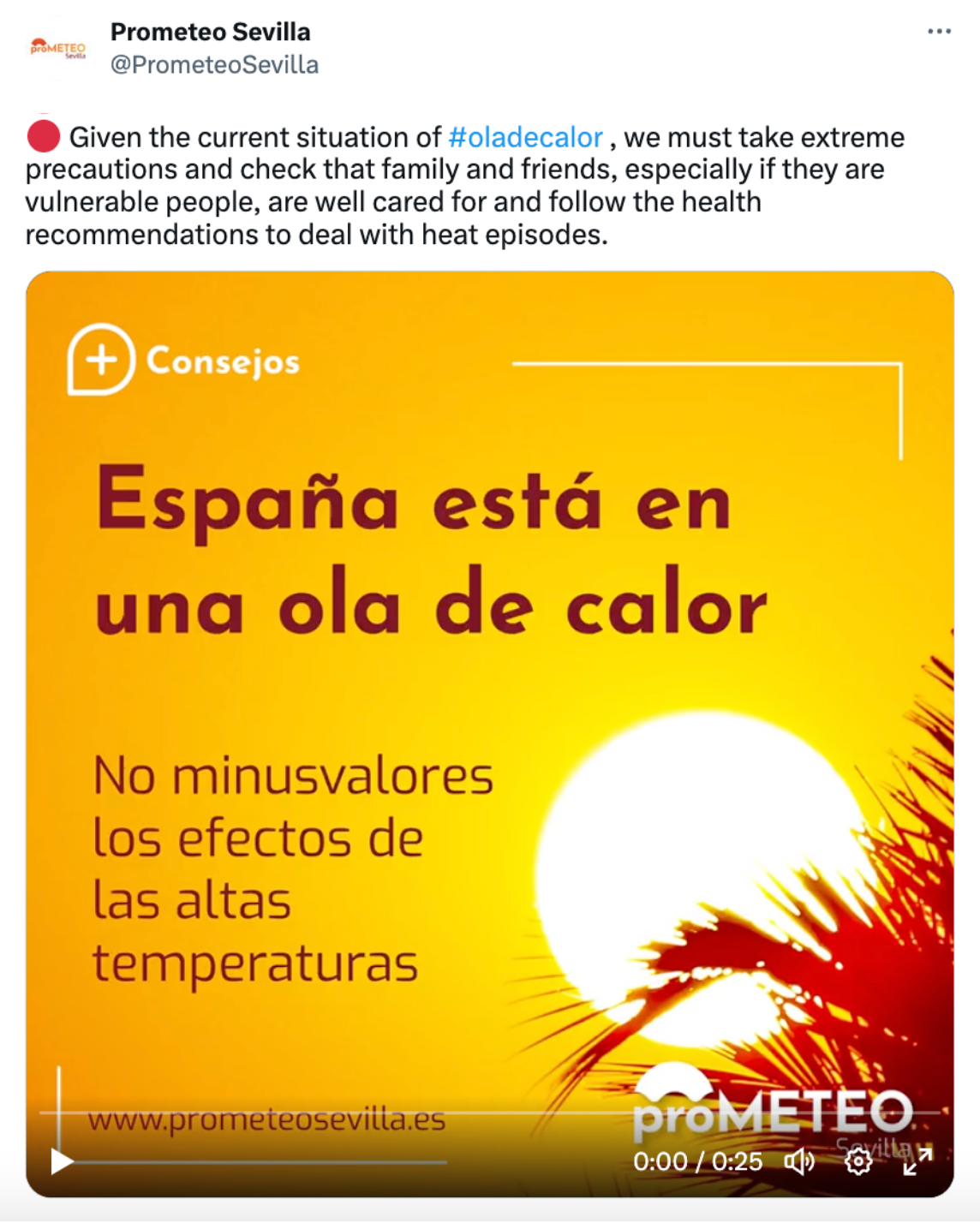
**

**
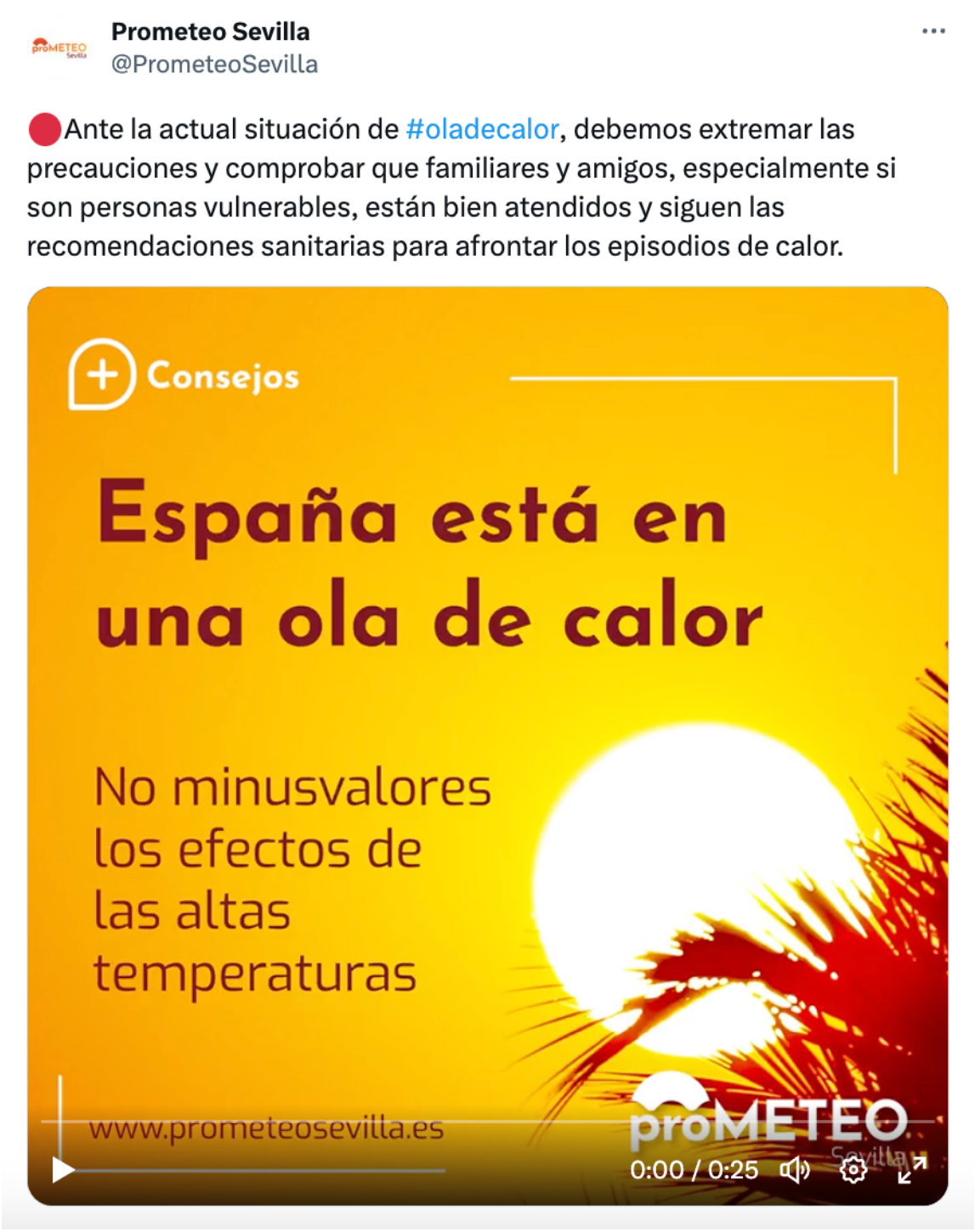
**

- [**Reduced impact of Zoe post**](https://twitter.com/PrometeoSevilla/status/1551871945276366849)

**
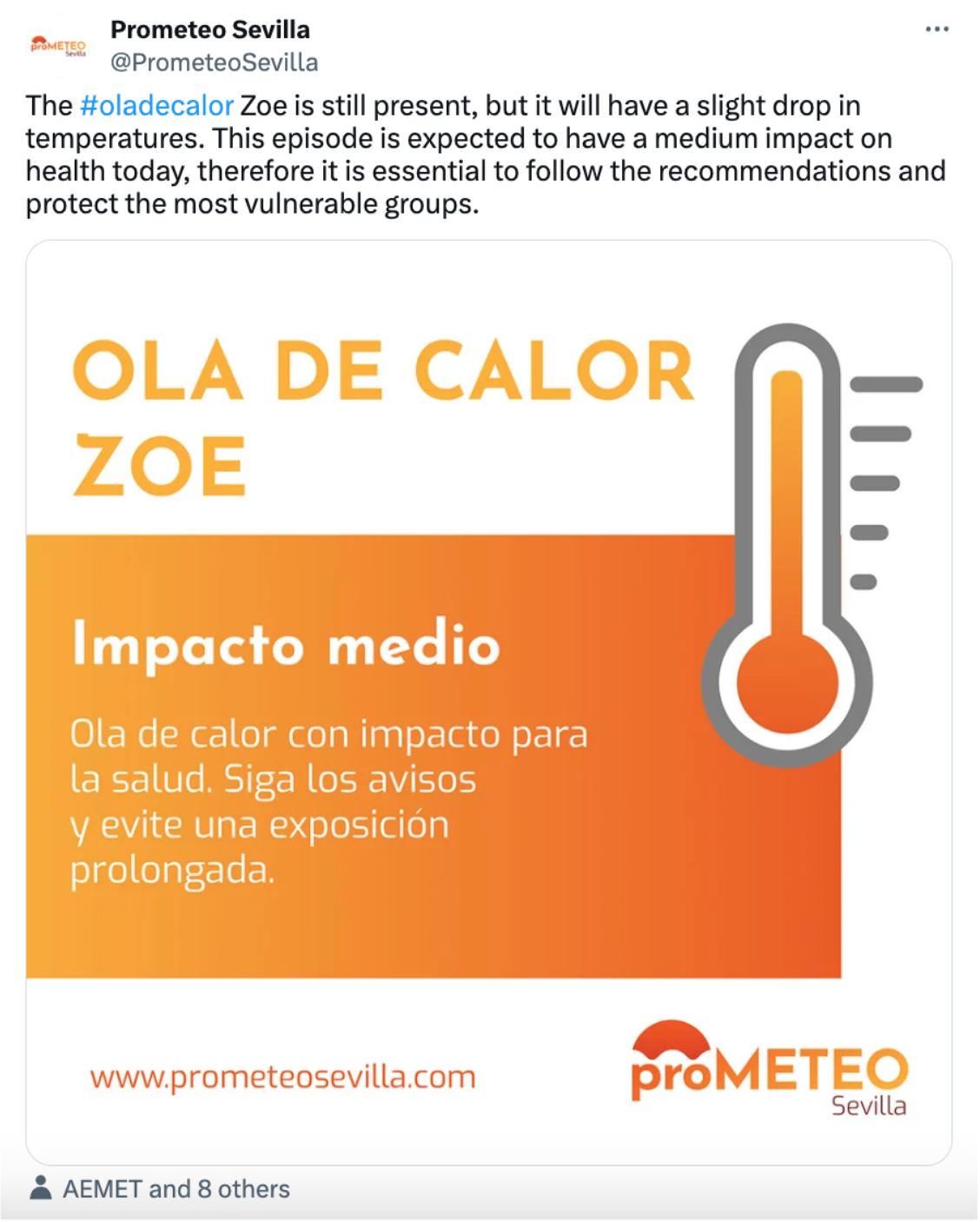
**

**
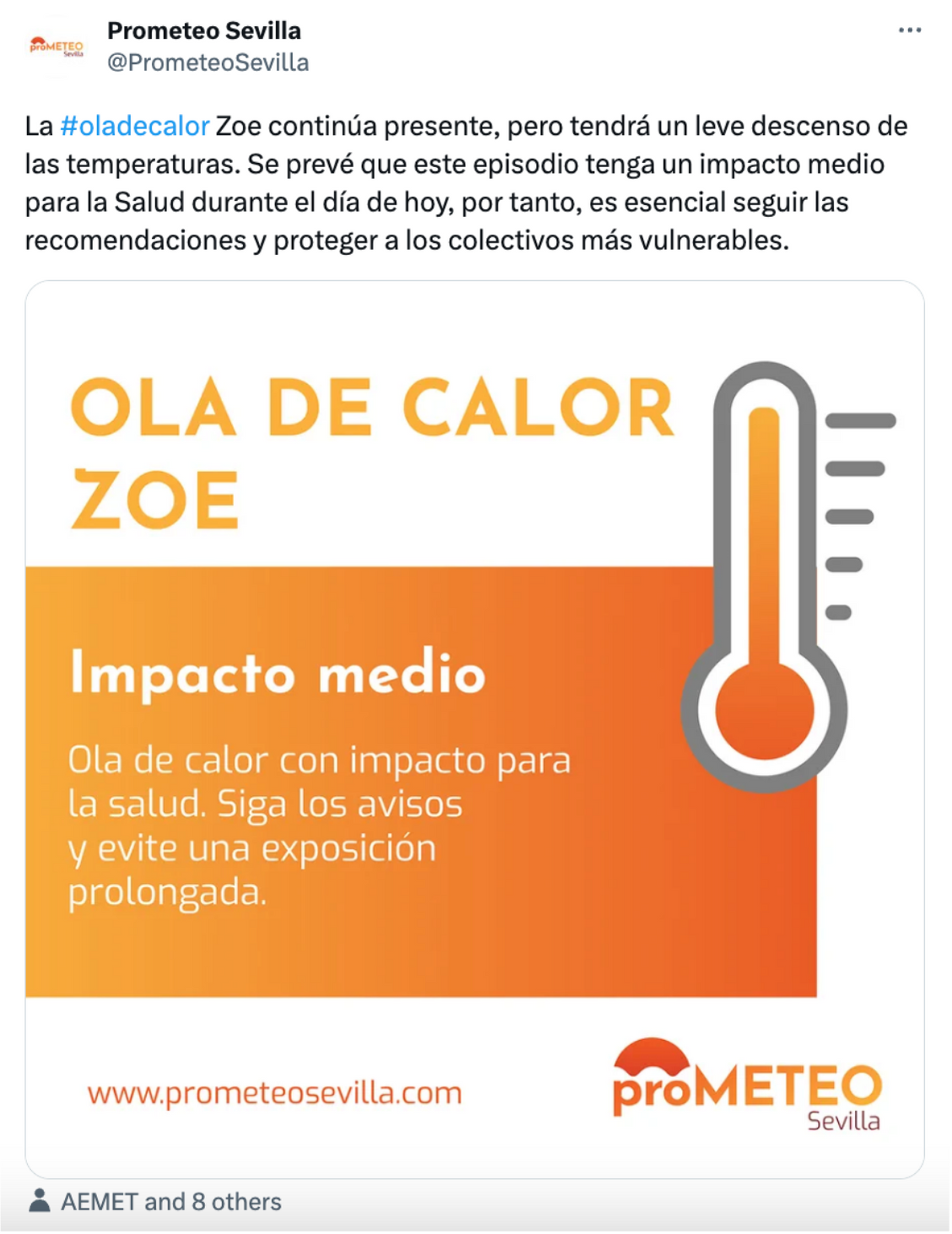
**

Complete survey (English translation)

[**Screening 1**](#_acd2qbew8hlc)

[**Awareness of Heat Waves 1**](#_mjbzctjltw6v)

[**Behavior 2**](#_eyt3lmdmdq56)

[**Awareness and Attitudes 3**](#_zb5tc9iz1kyv)

[**Demographics 4**](#_lkfjhqnyw5vt)

# Screening

1. How old are you? [###]
   1. [Terminate if under 25]
2. Where do you live?
   1. Almería [Terminate]
   2. Cádiz
   3. Córdoba
   4. Granada [Terminate]
   5. Huelva
   6. Jaén [Terminate]
   7. Málaga
   8. Sevilla
   9. Somewhere else [Terminate]

**Q2-ALT for comparison cities:**

Where do you live?

- 1. Badajoz
  2. Cáceres
  3. Salamanca
  4. Madrid
  5. Somewhere else [Terminate]

# Awareness of Heat Waves

1. How many heat wave warnings occurred in your area this summer? (If you’re not sure, take your best guess) [pull-down, None to 15]
2. Do you recall whether any of the heat waves this summer were categorized by level of severity?
   1. Yes
   2. No **[skip to Q6 if (b) is chosen]**
3. Do you recall what system was used?
   1. Levels 1 to 10
   2. Levels 0 to 3
   3. Levels A, B, and C
   4. Another system, please specify: [open end]
   5. I don’t recall
4. When was the last time you recall hearing about a heat wave warning in your area? [pull-down]
   1. Before May
   2. May
   3. June
   4. July
   5. August
   6. Earlier this month
   7. I have never heard a heat wave warning **[skip to Q12-ALT 3 Branch if (g) is chosen]**
5. How many, if any, of the heat waves this summer were named?
   1. None
   2. 1
   3. 2
   4. 3
   5. More than three

[If 1, go to Q8; If 2, 3 or more go to Q9; If “none” go to Q11-ALT2 branch]

1. [If Q7 =1] What was the name of that heat wave? [Open-end, **skip to Q10 if response does not contain “Zoe.” Otherwise, skip to Q11**]
2. [If Q7 = 2, 3 or “more”] What were the heat waves names you recall from this summer? [Open end, **skip to Q10 if response does not contain “Zoe.” Otherwise, skip to Q11**]
3. [No skip back from this point] Do you recall a heat wave named “Zoe” this summer?
   1. Yes [**Continue to Q11**]
   2. No [**Go to Q11-ALT 1 branch**]
4. When did you first learn about Heat Wave Zoe?

**Q11-ALT 1 Branch:** Thinking back to the **last named heat wave you recall**, when did you first learn it was a heat wave?

- 1. Two or more days before the heat wave began
  2. A day before the heat wave began
  3. The first day of the heat wave
  4. During the heat wave, but after the first day
  5. Only after the heat wave was over

**Q11-ALT 2 Branch:** Thinking back to the last heat wave you recall, when did you first learn it was a heat wave?

- 1. Two or more days before the heat wave began
  2. A day before the heat wave began
  3. The first day of the heat wave
  4. During the heat wave, but after the first day
  5. Only after the heat wave was over

# Behavior

1. Which, if any of the following, did you do during that heat wave? Check all that apply. [randomize order]

[Q12-**ALT 3 Branch**: Which of the following did you do the last time you experienced extremely hot temperatures this summer?]

- 1. Spent more time outdoors
  2. Spent more time indoors
  3. Changed my leisure plans to avoid the high heat
  4. Changed my work hours to avoid the high heat
  5. Worked from home to avoid the high heat
  6. Helped someone else avoid the high heat
  7. Drank more water than I usually do
  8. Warned others about the heat wave
  9. Told others how to stay safe in the high heat
  10. Found a place outside my home to stay cool
  11. More closely followed the forecast
  12. Dressed differently to protect myself from the heat
  13. Changed what I eat
  14. I did nothing differently than I usually do

1. Four typical glasses of water equal one liter. Roughly how many glasses of water did you drink on an average day during that heat wave? [##]
2. How many glasses of water do you drink on an average day when there is no heat wave? [##]
3. During **Heat Wave Zoe**, how often were you able to change your plans to avoid the high heat?
   1. Never able to avoid the high heat
   2. Sometimes able to avoid the high heat
   3. Able to avoid the high heat about half the time
   4. Able to avoid the high heat most of the time
   5. Always able to avoid the high heat

**ALT 1 or 2 Branch [If Q10 is (b)]**: During the **most recent heat wave**, how often were you able to change your plans to avoid the high heat?

**ALT 3 Branch [If Q6 is (g)]**: The last time you experienced extremely high temperatures, how often were you able to change your plans to avoid the high heat?

# Awareness and Attitudes

1. Healthy people do not need to alter their routine during a heat wave.
   1. Strongly agree
   2. Somewhat agree
   3. Neither agree or disagree
   4. Somewhat disagree
   5. Strongly disagree
2. Spending time outdoors during a heat wave can put me at a significant risk of suffering a heat stroke.
   1. Strongly agree
   2. Somewhat agree
   3. Neither agree or disagree
   4. Somewhat disagree
   5. Strongly disagree
3. I know what to do to stay safe during a heat wave.
   1. Strongly agree
   2. Somewhat agree
   3. Neither agree or disagree
   4. Somewhat disagree
   5. Strongly disagree
4. I know how to help others during a heat wave.
   1. Strongly agree
   2. Somewhat agree
   3. Neither agree or disagree
   4. Somewhat disagree
   5. Strongly disagree
5. Most people alter their routine to ensure their own safety during a heat wave.
   1. Strongly agree
   2. Somewhat agree
   3. Neither agree or disagree
   4. Somewhat disagree
   5. Strongly disagree
6. I am not the kind of person who needs to worry about heat waves
   1. Strongly agree
   2. Somewhat agree
   3. Neither agree or disagree
   4. Somewhat disagree
   5. Strongly disagree
7. Named weather events are more dangerous on average than unnamed ones
   1. Strongly agree
   2. Somewhat agree
   3. Neither agree or disagree
   4. Somewhat disagree
   5. Strongly disagree
8. The local government is effectively working to protect me from heat waves.
   1. Strongly agree
   2. Somewhat agree
   3. Neither agree or disagree
   4. Somewhat disagree
   5. Strongly disagree
9. I have easy access to a cool place when I need it in a heat wave.
   1. Strongly agree
   2. Somewhat agree
   3. Neither agree or disagree
   4. Somewhat disagree
   5. Strongly disagree
10. I support the idea of naming heat waves.
    1. Strongly agree
    2. Somewhat agree
    3. Neither agree or disagree
    4. Somewhat disagree
    5. Strongly disagree

# Demographics

1. Which of the following best describes your gender?
   1. Male
   2. Female
   3. Non-binary / third gender
   4. Prefer not to say
   5. Another gender not listed here
2. In the last twelve months, would you say that your state of health has been very good, good, fair, bad, very bad?
   1. Very good
   2. Good
   3. Fair
   4. Bad
   5. Very Bad
3. Are you experiencing any of the following? (Select all that apply).
   1. Heart conditions or coronary disease
   2. Respiratory problems (such as asthma and lung disease)
   3. Hypertension
   4. Diabetes
   5. Pregnancy
   6. Mental illness
   7. Drug or alcohol addiction
   8. None of these
4. Where do you work most of the time?
   1. Indoors, in a place that is cool
   2. Indoors in a place that is warm
   3. Outdoors, in a shaded area
   4. Outdoors, without shade
5. Which of the following do you have in your home or apartment? (Select all that apply)
   1. Air conditioning
   2. Ceiling fans
   3. Window fans
   4. Other fans
   5. Shades or curtains to block the sun
   6. Shaded film on the windows

**[Continue if (a) is chosen; skip to Q33 if (a) is not chosen]**

1. How often do you decide not to operate your air conditioning on hot days?
   1. I have stopped using air conditioning completely.
   2. I don’t use air conditioning on most hot days, even though I need it.
   3. I don’t use air conditioning on some hot days, even though I need it.
   4. I use my air conditioning on hot days whenever I need it. **[skip to Q33]**
2. Which of the following best describes the primary reason that you do not use air conditioning in your home?
   1. Cost
   2. Health concerns
   3. I don’t feel that I need it
   4. Other, please specify: [open end]
3. Which of the following intervals best represents the net monthly income of your entire household, after deductions for taxes, Social Security, etc.?
   1. Less than 570 euros
   2. From 570 to less than 800 euros
   3. From 800 to less than 1050 euros
   4. From 1,050 to less than 1,300 euros
   5. From 1,300 to less than 1,550 euros
   6. From 1,550 to less than 1,800 euros
   7. From 1,800 to less than 2,200 euros
   8. From 2,200 to less than 2,700 euros
   9. From 2,700 to less than 3,600 euros
   10. From 3,600 to less than 4,500 euros
   11. From 4,500 to less than 6,000 euros
   12. From 6,000 euros onwards

Complete survey: Spanish translation

[**Cribado 1**](#_acd2qbew8hlc)

[**Conciencia Sobre las Olas de Calor 1**](#_mjbzctjltw6v)

[**Comportamiento 2**](#_eyt3lmdmdq56)

[**Conciencia y Actitudes 2**](#_zb5tc9iz1kyv)

[**Demografía 3**](#_lkfjhqnyw5vt)

# Cribado

1. ¿Qué edad tiene?
   1. [Terminar si es menos de 25 años]
2. ¿En qué ciudad vive?
   1. Almería [Terminar]
   2. Cádiz
   3. Córdoba
   4. Granada [Terminar]
   5. Huelva
   6. Jaén [Terminar]
   7. Málaga
   8. Sevilla
   9. Otra ciudad [Terminar]

# Conciencia Sobre las Olas de Calor

1. Recuerde el pasado verano, ¿cuántas alertas de ola de calor tuvieron lugar en su zona? (Si no está seguro o no recuerda, indique un número que crea sea el más acertado). [seleccione, Ninguna a 15]
   1. #4 -ALT (solamente para aquellos que respondieron “c” a la pregunta #3) – ¿Cuándo se mudó a Andalucía? [Mes / Año]
2. ¿Cuándo fue la primera vez que recuerda una alerta de ola de calor en su zona? [seleccione]
   1. Antes de 2021
   2. Mayo de 2021
   3. Junio de 2021
   4. Julio de 2021
   5. Agosto de 2021
   6. Septiembre de 2021
   7. A comienzos de este año
   8. Nunca he escuchado ~~sobre~~ una advertencia de ola de calor [saltar #6, preguntar ALT-#7 en cambio]
3. Durante el verano pasado, ¿recuerda si alguna ola de calor tenía un nombre?
   1. [si responde “sí”] ¿Cuál era el nombre de la ola de calor que recuerda?
4. Recuerde la última ola de calor que vivió, ¿cuándo supo que se trataba realmente de una ola de calor?
   1. Dos o más días antes de que la ola de calor comenzara
   2. Un día antes de que la ola de calor comenzara
   3. El primer día de la ola de calor
   4. Durante la ola de calor, pero después del primer día
   5. Después de que la ola de calor terminara

# Comportamiento

1. ¿Qué hizo durante esa ola de calor? Seleccione todas las opciones que sean válidas [orden aleatorio]

[ALT-#7: ¿Qué hizo la última vez que enfrentó una ola de calor o temperaturas altas extremas?]

- 1. Pasé más tiempo al aire libre
  2. Pasé más tiempo en casa o dentro de edificaciones
  3. Cambié mis planes de ocio para evitar el calor extremo
  4. Cambié mis horas de trabajo para evitar el calor extremo
  5. Trabajé desde mi casa para evitar el calor extremo
  6. Ayudé a alguien a evitar el calor extremo
  7. Tomé más agua de la que normalmente tomo
  8. Advertí a otras personas sobre el calor extremo
  9. Hablé con otros sobre cómo protegerse del calor extremo
  10. Encontré un lugar fuera de mi casa para poder refrescarme
  11. Presté más atención a los previsiones del clima
  12. Me vestí un poco diferente para poder protegerme del calor
  13. Cambié lo que comí durante esos días
  14. En realidad no hice nada diferente a lo que hago todos los días

1. Unos cuatro vasos de agua equivalen a un litro. Más o menos, ¿cuántos vasos de agua tomó cada día durante la última ola de calor? [##]
2. ¿Cuántos vasos de agua toma de promedio cada día cuando no hay una ola de calor en su área? [##]
3. Durante la ola de calor más intensa del pasado verano, ¿con qué frecuencia pudo cambiar sus planes para poder evitar el calor extremo?
   1. Nunca pude evitar el calor extremo
   2. Algunas veces pude evitar el calor extremo
   3. Pude evitar el calor extremo la mitad de las veces
   4. Pude evitar el calor extremo casi todas las veces
   5. Siempre pude evitar el calor extremo

# Conciencia y Actitudes

1. Las personas saludables no necesitan cambiar sus rutinas diarias durante una ola de calor.
   1. Completamente de acuerdo
   2. Parcialmente de acuerdo
   3. Ni de acuerdo ni en desacuerdo
   4. Parcialmente en desacuerdo
   5. Completamente en desacuerdo
2. Pasar más tiempo al aire libre durante una ola de calor me coloca en una situación de mayor riesgo de sufrir un golpe de calor.
   1. Completamente de acuerdo
   2. Parcialmente de acuerdo
   3. Ni de acuerdo ni en desacuerdo
   4. Parcialmente en desacuerdo
   5. Completamente en desacuerdo
3. Sé bien qué hacer para protegerme durante una ola de calor.
   1. Completamente de acuerdo
   2. Parcialmente de acuerdo
   3. Ni de acuerdo ni en desacuerdo
   4. Parcialmente en desacuerdo
   5. Completamente en desacuerdo
4. Sé cómo ayudar a otros para protegerse durante una ola de calor.
   1. Completamente de acuerdo
   2. Parcialmente de acuerdo
   3. Ni de acuerdo ni en desacuerdo
   4. Parcialmente en desacuerdo
   5. Completamente en desacuerdo
5. La mayoría de las personas cambian sus rutinas diarias para protegerse durante una ola de calor.
   1. Completamente de acuerdo
   2. Parcialmente de acuerdo
   3. Ni de acuerdo ni en desacuerdo
   4. Parcialmente en desacuerdo
   5. Completamente en desacuerdo
6. Soy una persona que no necesita preocuparse en caso de una ola de calor.
   1. Completamente de acuerdo
   2. Parcialmente de acuerdo
   3. Ni de acuerdo ni en desacuerdo
   4. Parcialmente en desacuerdo
   5. Completamente en desacuerdo

# Demografía

1. ¿Cuál es su género?
   1. Hombre
   2. Mujer
   3. Prefiero no responder
2. En los últimos 12 meses, ¿cómo diría que ha sido su estado de salud?
   1. Muy bueno
   2. Bueno
   3. Tolerable
   4. Malo
   5. Muy malo
3. ¿Sufre o ha sufrido alguno de los siguientes problemas de salud? (Seleccione todas las opciones válidas).
   1. Problemas del corazón o enfermedades coronarias
   2. Problemas respiratorios (como asma o enfermedades en los pulmones)
   3. Hipertensión
   4. Diabetes
   5. Enfermedades mentales
   6. Adicción al alcohol o a drogas
   7. Ninguna de las anteriores
4. ¿Dónde trabaja la mayoría de su tiempo?
   1. En una oficina
   2. En mi casa
   3. Al aire libre
   4. Dentro o fuera de casa u oficina, dependiendo del día
   5. En otra parte. Por favor, describa: [abierto]
5. ¿Cuáles de los siguientes medios para evitar el calor tiene en su casa? (Seleccione todas las opciones válidas)
   1. Aire acondicionado
   2. Ventiladores de techo
   3. Otra clase de ventiladores
   4. Cortinas opacas para bloquear el sol
   5. Cristales tintados
   6. Las persianas
6. ¿Con qué frecuencia decide no encender su aire acondicionado durante días calurosos por el alto precio de la electricidad?
   1. Yo he dejado de usar completamente el aire acondicionado debido a los costes
   2. Yo no uso aire acondicionado la mayoría de los días muy calurosos, a pesar de que lo necesito, debido al alto precio de la electricidad.
   3. Yo no uso aire acondicionado en algunos días muy calurosos, a pesar de que lo
   4. necesito, debido al alto precio de la electricidad.
   5. Yo uso mi aire acondicionado en los días muy calurosos a pesar de que puede resultar muy costoso
7. ¿Cuál de las siguientes opciones representa mejor su ingreso familiar mensual neto? (Incluyendo a todos los miembros de la familia y tras las deducciones de impuestos, Seguro Social y otros)
   1. Menos de 570 euros
   2. Entre 570 y 800 euros
   3. Entre 800 y 1.050 euros
   4. Entre 1.050 y 1.300 euros
   5. Entre 1.300 y 1.550 euros
   6. Entre 1.550 y 1.800 euros
   7. Entre 1.800 y 2.200 euros
   8. Entre 2.200 y 2.700 euros
   9. Entre 2.700 y 3.600 euros
   10. Entre 3.600 y 4.500 euros
   11. Entre 4.500 y 6.000 euros
   12. Más de 6.000 euros
